# B cell αv integrin regulates tissue specialization and clonal expansion of lung germinal center and memory B cells after viral infection

**DOI:** 10.1126/sciadv.aeb7633

**Published:** 2026-06-12

**Authors:** Andrea Montiel-Armendariz, Kelsey Roe, Jonathan Lagos, Laura Veronica Martinez-Castro, Matthew J. Dufort, Oliver J. Harrison, Adam Lacy-Hulbert, Mridu Acharya

**Affiliations:** ^1^ Center for Immunity and Immunotherapies, Seattle Children’s Research Institute, Seattle, WA, USA.; ^2^ Center for Systems Immunology, Benaroya Research Institute, Seattle, WA, USA.; ^3^ Center for Fundamental Immunology, Benaroya Research Institute, Seattle, WA, USA.; ^4^ Department of Immunology, University of Washington, Seattle WA, USA.; ^5^ Department of Pediatrics, University of Washington, Seattle, WA, USA.

## Abstract

Lung-resident B cells are increasingly recognized as key contributors to protective immunity against respiratory viruses, yet the mechanisms that govern their generation and specialization remain poorly understood. Here, we identify B cell–intrinsic αv integrin as a critical negative regulator of germinal center (GC) dynamics and memory B cell formation in the lung following influenza A virus infection. Using B cell–specific αv knockout mice, we show that loss of B cell αv integrin leads to persistent GC activity within the inducible bronchus-associated lymphoid tissue and expansion of lung-resident memory B cells, including IgA^+^ and cross-reactive B cells capable of recognizing heterologous influenza variants. Single-cell transcriptomic and B cell receptor sequence analyses reveal that αv restricts clonal expansion and antigenic diversification of GC and memory B cells in the lung, but not in draining lymph nodes, indicating a spatially restricted mechanism of mucosal B cell regulation. These findings position αv integrin as a key checkpoint that constrains local mucosal B cell evolution and suggest previously unexplored strategies to improve mucosal vaccine efficacy by enhancing GC activity directly in the lung.

## Teaser

αv integrin regulates lung-resident B cell responses during respiratory viral infections.

## INTRODUCTION

Protective immunity against rapidly mutating pathogens such as respiratory viruses, HIV, and malaria depends on the ability of B cells to generate antibodies with high affinity and broad specificities as well as the long-term protection provided by memory B cells and long-lived plasma cells. While these features of B cell responses have been extensively studied in the context of systemic immune responses, recent studies are revealing the importance of localized B cell responses in mucosal tissues, particularly the lung, in combating respiratory pathogens.

Following influenza infection, early extrafollicular B cell responses are known to contribute to viral clearance (*1*), whereas lung-resident memory B cells are known to provide critical protection against secondary infection by producing broadly reactive and often immunoglobulin A (IgA)–class-switched antibodies at mucosal sites (*2*–*4*). IgA antibody, in particular, offers advantages due to its polymeric structure and the ability to neutralize pathogens at epithelial barriers. However, despite growing evidence of their protective function, the mechanisms that control the generation and maintenance of these lung-resident B cells are still poorly understood. Moreover, these lung-resident B cells are not effectively induced by systemic vaccination strategies. Therefore, understanding the regulatory mechanisms behind lung-resident B cell responses is essential for developing vaccines that provide durable mucosal immunity against respiratory pathogens.

We previously identified a regulatory role for the αv integrin family in systemic B cell responses. αv integrins play diverse roles in multiple immune cell populations, such as dendritic cells, macrophages, T cells, and B cells (*5*–*12*). We described a new function for αv integrin in regulating B cell responses to Toll-like receptor (TLR) ligands and TLR ligand–containing antigens, via regulation of intracellular trafficking events (*13*). Mechanistically, αv integrin engages autophagy-related proteins to regulate the intracellular trafficking of TLRs and their ligands, thereby modulating the magnitude and duration of TLR signaling in B cells. Therefore, B cells lacking αv integrin show increased and persistent TLR signaling resulting in increased B cell responses to TLR ligands (*13*, *14*). While αv-mediated regulation of TLR signaling serves to limit autoreactive responses in autoimmune settings (*15*), this same pathway also constrains protective immunity. Mice lacking B cell αv integrins develop heightened germinal center (GC) responses and show increased somatic hypermutation (SHM) of antibodies following systemic immunization with TLR ligand–containing antigens (*16*). B cell–specific αv knockout mice also showed better protection after infection with influenza virus (*16*). However, it remained unclear whether this integrin-TLR axis influences B cell responses in mucosal tissues, where local inflammatory cues and persistent antigen exposure may uniquely shape immune regulation. On the basis of the known role of TLR signaling in mucosal B cell activation and the heightened presence of TLR ligands in inflamed lungs during infection, we hypothesized that B cell–intrinsic αv integrins may restrain local B cell responses at the respiratory tract.

To test this, we used a mouse model of intranasal influenza A virus (IAV) infection in combination with B cell–specific αv deletion, single-cell RNA sequencing, antigen-specific B cell tracking, and clonal lineage analysis. This approach allowed us to uncover a previously unrecognized, tissue-restricted role for αv integrin in regulating lung GC persistence, clonal expansion of B cells in the lungs, and the emergence of IgA^+^ and cross-reactive memory B cells. These findings reveal αv integrin as a key checkpoint that constrains mucosal B cell evolution and suggest previously unexplored avenues for enhancing lung-resident humoral immunity through targeted modulation of local GC activity.

## RESULTS

### B cell αv integrin regulates generation of cross-reactive lung-resident GC and memory B cells following influenza infection

**Fig. 1.**
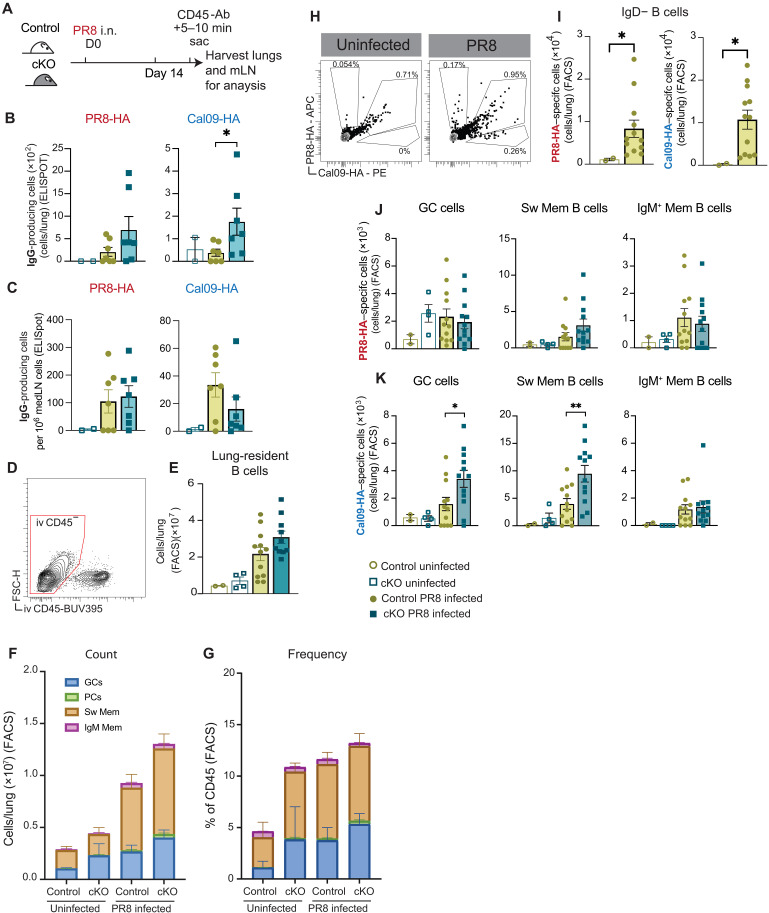
B cell αv integrin regulates cross-reactive B cell responses in the lungs. αv^+/+^ CD19^Cre+^ (control) and αv^fl/fl^ CD19^Cre+^ (cKO) mice were infected intranasally (i.n.) with low dose of live PR8 IAV. Fourteen days later, mice received a retro-orbital (r.o.) injection with 1 μg of anti-CD45 antibody (Ab) (α-CD45-BUV395) 5 min before euthanasia. (**A**) Schematic for experimental procedure. Ab, antibody; D0, day 0. (**B** and **C**) Quantification of IgG-secreting cells in the lungs (B) or mediastinal lymph node (medLN) (C) that recognize PR8-HA or Cal09-HA as identified by ELISpot. (**D**) Representative flow cytometry plot of the intravenous CD45 staining in the lungs. (**E**) Quantification of live B cells gated as lung-resident [intravenous (iv) CD45-unlabeled] B cells (CD19^+^ CD138^+/−^). (**F** and **G**) Count (F) and frequency (% of intravenous CD45-unlabeled cells) (G) of B cell subpopulations. Lung-resident B cells gated into subpopulations based on the surface markers: Plasma cells (PCs) gated as IgD^−^ IRF4^+^ CD138^+^ cells, germinal center cells (GCs) gated as IgD^−^ IRF4^−^ CD138^−^ Fas^+^ PNA^+^, and switched (Sw) memory (Mem) phenotype B cells gated as IgD^−^ IRF4^−^ CD138^−^ Fas^−^ PNA^−^ IgM^−^ IgA^−/+^ (see fig. S1 for gating strategy). Cells per lung were calculated by multiplying frequency of each population (% of live cells), determined by flow cytometry, by the number of live cells recovered from the tissue. (**H**) Representative flow plot of frequency of PR8-HA or Cal09-HA tetramer^+^ cells in an uninfected (left) and infected mouse (right) on lung-resident IgD^−^ B cells. (**I**) Quantification of number of lung-resident IgD^−^ B cells specific for PR8-HA or Cal09-HA. (**J** and **K**) Quantification of lung-resident PR8-HA–specific (J) or Cal09-HA–specific (K) GCs, Sw memory B cells (Sw Mem B cells) and IgM^+^ memory B cells (IgM^+^ Mem B cells). Each dot represents individual mouse (*n* = 2 to 4 mice uninfected and *n* = 7 to 12 mice infected). Data are means ± SEM of either combination of two independent experiments (B to I) or a representative experiment (J and K). *P < 0.05 and **P < 0.01 by Mann-Whitney *U* test. FSC-H, forward scatter-height.

To investigate whether B cell–intrinsic αv integrin regulates tissue-specific B cell responses during respiratory viral infection, we intranasally infected B cell–specific αv conditional knockout mice (referred to as cKO mice) and matched controls with a sublethal dose of H1N1 PR8 IAV (Fig. 1A). Fourteen days postinfection, we used enzyme-linked immunospot (ELISpot) assay to identify overall changes in antigen-specific B cells from lung and draining mediastinal lymph nodes (medLNs). Compared with controls, lungs from cKO mice showed increased numbers of PR8-HA–specific IgG-secreting B cells after infection (Fig. 1B). Moreover, Cal09-HA–specific IgG-secreting cells were significantly increased in cKO lungs compared with controls (Fig. 1B). In contrast, hemagglutinin (HA)–specific antibody-secreting cells in the medLN were variable and did not differ significantly between genotypes, with no consistent enhancement in cKO mice (Fig. 1C).

To determine which B cell subsets accounted for these tissue-specific changes, we used flow cytometry with intravenous labeling to distinguish circulating B cells from lung-resident B cells and gated on different B cell subpopulations (fig. S1 and Fig. 1D). Total numbers of intravenous labeling ^neg^ lung-resident B cells increased equivalently postinfection in both genotypes (Fig. 1E), and this increase was largely driven by memory B cells accumulation (Fig. 1, F and G). We also identified intravenous labeling ^neg^ lung-resident GC-like B cells by surface expression of FAS^+^ PNA^+^, which were present at higher baseline levels in cKO lungs, consistent with our prior observations of increased spontaneous GCs in Peyer’s patches (*16*). Although both genotypes showed an increase in these lung GC-like B cells after infection, αv-deficient mice exhibited higher GC-like numbers at baseline and sustained elevated levels following infection (Fig. 1, F and G), suggesting that αv could influence the amplitude or persistence of lung GC responses.

We next evaluated the antigen specificity and cross-reactivity of the lung-resident B cell subpopulation using HA tetramers from PR8 and Cal09 IAV strains (*17*). After verifying that these tetramers could reliably identify either lung-resident PR8-HA^+^ or Cal09-HA^+^ B cells induced by PR8 infection (Fig. 1, H and I), we compared HA-specific lung-resident B cells in the PR8 infected control and cKO mice. The number of infection-induced PR8-HA–specific lung-resident GC B cells was not significantly altered between the genotypes, although class-switched memory phenotype B cells were slightly increased in cKO lungs (Fig. 1J). In contrast, infection-induced lung-resident Cal09-HA–binding B cells of GC and memory phenotype were significantly elevated in cKO lungs compared with controls (Fig. 1K), indicating enhanced cross-reactive responses in the absence of αv. Furthermore, lung-resident IgM^+^ memory B cells specific for either PR8-HA or Cal09-HA were not increased in cKO lungs, suggesting that αv deficiency preferentially enhances class-switched antigen-experienced B cell subsets (Fig. 1, J and K). These differences were not observed in the medLN, where HA-specific GC and memory B cell frequencies or numbers were low and did not show significant differences between groups (fig. S2, A to F), reinforcing the tissue-restricted effect of B cell αv loss in promoting B cell responses after intranasal infection.

These findings demonstrate that B cell–intrinsic αv integrin specifically restrains the generation of cross-reactive, lung-resident, GC, and memory B cells following influenza infection.

### Loss of B cell αv integrin increases lung-resident IgA^+^ B cells following influenza virus infection

Secretory IgA plays a key role in mucosal defense against respiratory viruses, but the mechanisms governing generation of IgA–class-switched B cells in the lung remain poorly understood. On the basis of the enhanced lung B cell responses seen in cKO mice, we examined whether B cell–intrinsic αv integrin influences the generation of IgA^+^ B cells following influenza infection.

**Fig. 2.**
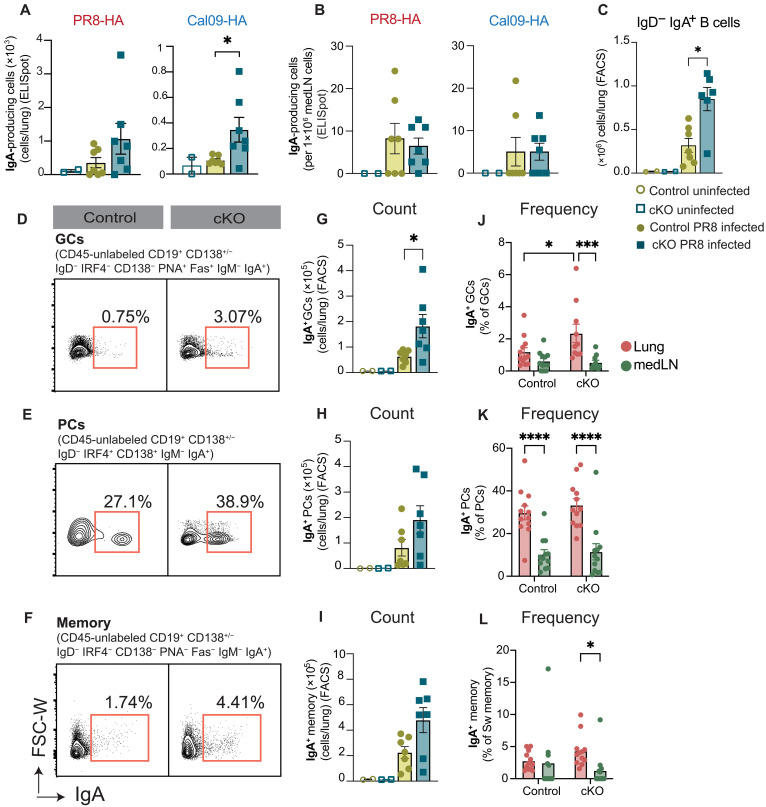
Absence of B cell αv integrin promotes IgA B cell response in the lungs after influenza infection. αv^+/+^ CD19^Cre+^ (control) and αv^fl/fl^ CD19^Cre+^ (cKO) mice were infected i.n. with low dose of live H1N1 PR8 IAV. After 14 days of infection, all mice received r.o. injection with 1 μg of α-CD45-BUV395 5 min before euthanasia. (**A** and **B**) IgA-secreting cells in the lungs (A) or medLN (B) that recognize PR8-HA (left) or Cal09-HA (right) as detected by ELISpot. (**C**) Quantification of lung-resident IgD^−^ IgA^+^ B cells by flow cytometry. (**D** to **F**) Representative flow plots of the expression of IgA on GC B cells (D), PCs (E), and memory B cells (F) (see fig. S1 for gating strategies). (**G** to **I**) Quantification from flow analysis of IgA^+^ GCs (G), PCs (H), and IgA memory B cells (I). (**J** to **L**) Quantification of the frequency of IgA^+^ GCs (J), PCs (K), and memory (L) as a frequency of their respective parent population in lungs and medLN. Each dot represents an individual mouse (*n* = 2 mice for uninfected group and *n* = 6 to 7 mice for infected groups). Data are means ± SEM from one representative experiment from three independent experiments (A to I) or combined from two experiments (J to L). *P < 0.05, ***P < 0.001, and ****P < 0.0001 by Mann-Whitney *U* test between the two PR8-infected groups (A to I) or two-way analysis of variance (ANOVA) comparing tissue and group (J to L). FSC-W, forward scatter width.

ELISpot analysis of HA-specific cells showed that, at day 14 postinfection, both cKO and control mice generated HA-reactive IgA-producing cells in the lungs (Fig. 2A). Anti–PR8-HA IgA-producing cells were present at higher numbers in some cKO mice compared with controls, but this did not reach statistical significance. However, Cal09-HA–binding cells, indicative of cross-reactive responses, were more consistently increased in cKO mice compared with controls, similar to our findings for IgG-producing cells (Fig. 2A). In contrast to the lung, the number of HA-specific IgA-secreting cells was similar between groups in the medLN (Fig. 2B), suggesting that B cell αv deficiency specifically enhances lung-localized IgA responses. Additional analysis of antibody-secreting cells in the medLN and lungs at day 7 postinfection further showed that there was no significant increase in either IgA- or IgG-secreting cells in cKO mice in the medLN at early time points (fig. S2, G and H).

To characterize these IgA^+^ B cells in the lungs, we performed flow cytometric phenotyping of lung-resident B cells. IgD^−^ IgA^+^ B cells were rare before infection in both genotypes but were robustly induced in the lungs following infection, with cKO mice displaying a ∼2-fold increase in total lung-resident IgA^+^ B cells compared with controls (Fig. 2C). This increase extended across the GC, plasma cell, and class-switched memory B cell compartments (Fig. 2, D to I), indicating that αv deficiency promotes IgA class switching related to multiple B cell subsets. In contrast, no significant genotype-dependent differences in IgD^−^ IgA^+^ B cells (frequency or numbers) were observed in the medLN (fig. S3A).

We next compared the distribution of IgA^+^ B cells between the medLN and lung tissue. While both sites showed infection-induced IgA^+^ B cells, there was a higher proportion of IgA^+^ cells within the lung-resident B cell compartments in both genotypes (Fig. 2, J to L). Moreover, the cKO mice showed a significant increase in the frequency of IgA^+^ cells within the lung-resident GC B cell compartment. Meanwhile the frequency (or numbers) of IgA^+^ B cells in the medLN remained low in both genotypes across all B cell compartments (Fig. 2, J to L, and fig. S3B). Consistent with this, comparison of IgA- versus IgG-secreting HA-specific cells in the lungs and medLN also showed that IgA-secreting cells were found predominantly in the lungs compared with the medLN and that the increase observed in cKO mice was predominantly localized to the lungs (fig. S3, C and D). Together, these data demonstrate that IgA^+^ B cells preferentially accumulate in the lung following influenza infection and that B cell–intrinsic αv integrin limits the generation of these cells, particularly those with a GC phenotype.

### Loss of B cell αv integrin promotes infection-induced GC reactions locally in the lungs

**Fig. 3.**
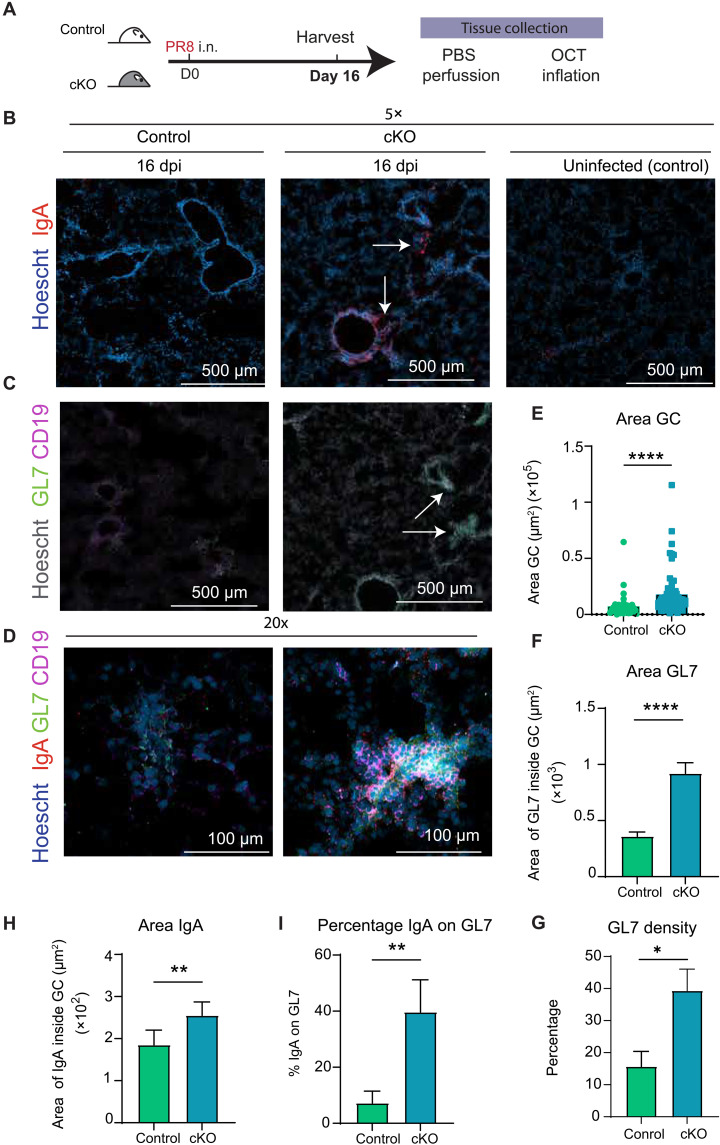
Loss of B cell αv integrin enhances the induction of iBALT-GCs in lungs after viral infection. αv^+/+^ CD19^Cre+^ (control) and αv^fl/fl^ CD19^Cre+^ (cKO) mice were infected i.n. with low dose of live PR8 IAV. Lungs were harvested after 16 days postinfection (dpi). (**A**) Schematic of mice infection and tissue collection for lung tissue staining. (**B** and **C**) Representative confocal images of lungs from uninfected control mouse and infected control or cKO mice. Images representing one single plane at 5× magnification. Scale bars, 500 μm. (B) Lung staining for Hoechst (nucleus; blue) or IgA (red). Airways surrounded by IgA staining are shown by arrows. (C) Lung staining for Hoechst (nucleus; gray), GL7 (green), and CD19 (magenta). Arrows represent GL7-positive and CD19-positive structures corresponding to iBALTs. (**D**) Representative confocal images of lungs from control or cKO mice 16 days postinfection. Images representing one single plane at 20× magnification labeled with Hoechst (nucleus; blue), IgA (red), GL7 (green), and CD19 (magenta) showing iBALTs. Scale bars, 100 μm. (**E**) Scatter plot with a bar graph showing the average size (in square micrometers) of GL7^+^CD19^+^ GC structures by manually quantifying the area of these structures. Every dot is the area of 1 GC structure (35 to 65 structures) from 22 to 28 different fields of sections viewed at 10× from three different mice per genotype. (**F**) Bar graph showing means and SEM of the area (in square micrometers) of GL7 staining within GCs. (**G**) GL7 density quantified as the frequency of GL7^+^ cells relative total CD19^+^ B cells in GC area. (**H**) IgA-positive staining inside the structures (*n* = 35 to 65) as defined in (E). (**I**) Bar graph showing means and SEM of the percentage of IgA^+^GL7^+^ staining within iBALT structures. Sections (*n* = 7 to 9) viewed at 20× from three different mice per group. *P < 0.05, **P < 0.01, and ****P < 0.0001 by Mann-Whitney *U* test.

To investigate whether the loss of B cell αv promotes lung-resident IgA^+^ B cells via local GC activity, we used confocal microscopy to examine lung sections from infected mice at day 16 postinfection (Fig. 3A). We confirmed that more IgA^+^ cells were present in cKO lungs compared with controls, and these cells were distributed around large airways and within clusters in the lung parenchyma (Fig. 3B and fig. S4A). We therefore asked whether these clusters of IgA^+^ cells were related to GC structures in the lungs.

Ectopic lung GC reactions have previously been shown to develop in inducible bronchus-associated lymphoid tissues (iBALTs) after influenza infection (*18*, *19*). Accordingly, we used CD19 staining to identify B cells and GL7 for GC B cells (Fig. 3C and fig. S5) and manually defined the regions where GL7^+^ B cells clustered together as the iBALT-GCs induced by infection. These iBALT-GC B areas were close to airways and found in the lungs of both control and cKO mice. Although the GC structures were not as abundant at this time point, as described by others at later time points (*20*), the cKO lungs exhibited larger and more organized clusters of GC B cells, whereas control lungs showed smaller, less organized GC clusters (Fig. 3, C and D). Quantification of either the manually gated iBALT-GC area (area GC) or the levels of GL7 staining within the GC regions showed that the clusters of GC cells were significantly larger in the lungs from cKO mice, compared with the lungs of the control mice (Fig. 3, E and F). An increase in the mean number of GCs per mouse lung was also observed, although the difference did not reach statistical significance (fig. S4B). We further used QuPath analysis to quantify the ratio of GL7^+^ cells to total B cells within the GC area. This analysis showed a significant increase in density of GL7^+^ cells in the cKO lungs compared with those in control (Fig. 3G), indicating that the increased GC areas in the cKO lungs are associated with increase in GC activity. In contrast, in the uninfected lungs, iBALT-GC areas were rare and inconsistently detected. Analysis of dispersed GL7^+^ B cells in the uninfected lungs showed minimal GL7^+^ B cells and no differences between genotypes, confirming that GC expansion is induced by infection (fig. S4C).

IgA^+^ cells were more frequently localized to iBALT-GCs in cKO lungs than in controls (Fig. 3H and fig. S4D), and IgA/GL7 colocalization was significantly higher in cKO sections (Fig. 3I). These imaging results were consistent with significantly increased numbers of IgA^+^ GC B cells observed by flow cytometry (Fig. 2, D and G). We next examined isotype composition within lung GCs and found that GC structures in control and cKO mice contained both IgG^+^ and IgA^+^ B cells (fig. S4, E and F). Direct comparison of IgG2c^+^ and IgA^+^ GC B cells at day 16 revealed a modest but reproducible increase in the proportion of IgA^+^ cells within cKO lung GCs (fig. S4G), suggesting that loss of αv may accelerate or favor IgA differentiation within lung GC responses. These findings show that loss of B cell αv integrin promotes the formation of ectopic GC structures directly in the lungs and thus enhances the generation of GC-derived IgA^+^ B cells following influenza infection.

### Loss of B cell αv leads to expansion of GC subclusters in the lungs and promotes cross-reactivity

The increase in the ectopic GC structures in the lungs of cKO mice raised questions about whether these GC cells were similar to canonical GC cells, such as those in the medLN. To address this question, we turned to single-cell RNA sequencing (scRNA-seq) of antigen-reactive B cells induced after infection. PR8-HA^+^ (antigen-specific) and Cal09-HA^+^ (cross-reactive) tissue-resident B cells were sorted from lungs and medLNs of cKO or control mice, 20 days after infection with H1N1 PR8. In these experiments, we made use of conformationally intact HA tetramers (*21*, *22*) rather than the linear HA probes used in Fig. 1, to more sensitively identify HA-specific B cells for analysis. We verified that these tetramers could be used to identify antigen-reactive cells by flow cytometry (fig. S8A).

**Fig. 4.**
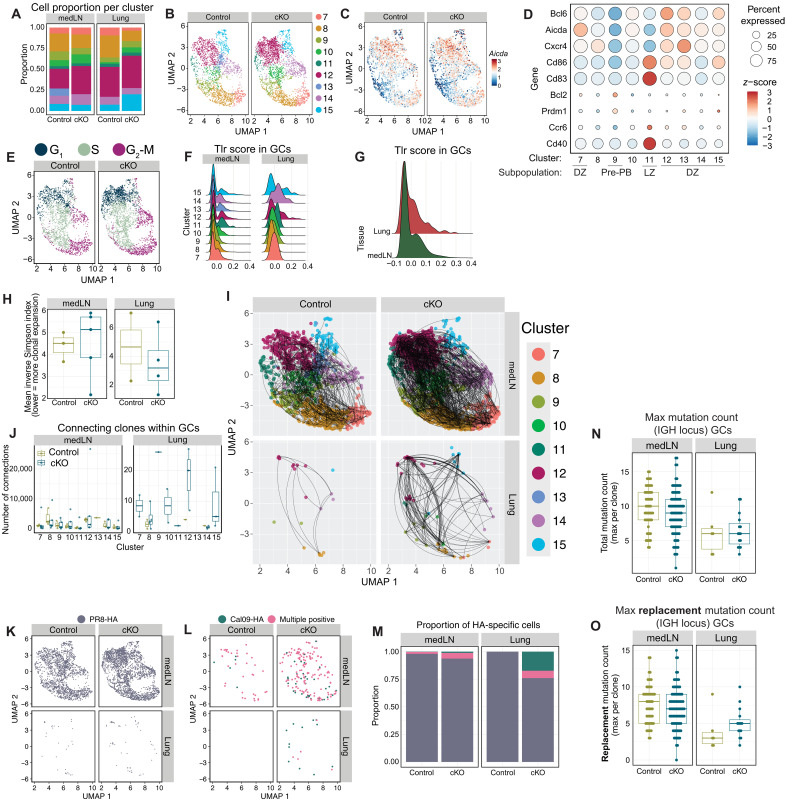
Loss of B cell αv leads to expansion of cross-reactive lung GC cells. scRNA-seq of sorted lung-resident or medLN PR8-HA^+^ or Cal09-HA^+^ B cells from control and cKO mice 20 days post–low-dose i.n. infection with live PR8. (**A** and **B**) GC subclusters identified by Louvain method. (A) Stacked bar graph of the proportion of cells in GC subclusters in lung and medLN. (B) UMAP of the GC subclusters in control and cKO mice. (**C**) Expression of *Aicda* in GCs. (**D**) Dot plot showing expression of key genes in GC subclusters; color represents the expression level (*z*-score), and circle size represents proportion of cells expressing that marker. (**E**) UMAP of the GC cells colored by cell cycle phase identified with Seurat. (**F** and **G**) Ridge plot of the *Tlr* score expression by cluster (F) or tissue (G). Score obtained from the Seurat package by calculating average expression of a gene set and subtracting aggregated expression of control gene sets. Gene set for *Tlr* score: *Tlr-1, Tlr-2, Tlr-3, Tlr-4, Tlr-6, Tlr-7*, and *Tlr-9*. (**H**) Inverse Simpson index of BCR clonal diversity; values are mean of repeated downsampling to a common number of GC cells per mouse in each tissue. (**I**) UMAP of GC subclusters showing BCR clonal relationships. Lines connect cells from the same BCR clone within each tissue; to illustrate differences, only 1/100 connections are shown. (**J**) Quantification of number of connecting lines of a clone to its sister clones within the GCs. Dots represent outliers. (**K** to **L**) UMAP of PR8-HA^+^ (K) and cross-reactive (L) (either Cal09-HA^+^ or multiple positive: binds to both PR8-HA and Cal09-HA) GC cells. (**M**) Quantification of proportion of cells by specificity in medLN and lungs. (**N** and **O**) Quantification of maximum number of total (N) and amino acid–replacing mutations (O) by clone. Each dot represents one clone.

For each B cell, we simultaneously obtained HA specificity (via oligo-tagged PR8-HA and Cal09-HA probes), B cell receptor (BCR) sequence from heavy and light chains, antibody isotype, and transcriptome with the 10x Chromium technology (fig. S6A). Clustering by Louvain community detection based on gene expression of all B cells identified 16 different clusters, which were assigned to one of four major cell types (naïve, GC, memory, and plasma cells) across both tissues based on the expression of *Ighd*, *Aicda*, *Cd80*, and *Prdm1* (fig. S6B). Deconvolution of oligo hash-tagged antibodies showed that some mice lacked antigen-specific GC B cells in the medLN, which indicated unsuccessful influenza infection. Consequently, cells from these uninfected mice were removed from downstream analysis. The remaining eight mice showed even representation of high-quality antigen-specific cells. We first focused on the GC clusters (clusters 7 to 15) and observed that lung-derived GC cells shared transcriptional similarities with those from the medLN. Despite being fewer in number, lung-derived GCs clustered together with comparable GC subtypes found in the medLN (fig. S6C). Notably, all GC subclusters were represented in both lung and medLN, with the exception of cluster 13, which was only present in the medLN (Fig. 4A and fig. S6D).

Subclustering of GC B cells (Fig. 4, A and B) identified expected subpopulations consistent with known GC biology, including subclusters showing higher *Aicda* expression (Fig. 4C). To further characterize the GC cells, we quantified expression of established dark zone (DZ)– and light zone (LZ)–associated gene sets across GC subclusters (*23*) using AUCell (*24*). Whereas cluster 7 showed high expression of characteristic DZ genes and cluster 11 had high expression of LZ genes, several other GC subclusters exhibited intermediate signatures (fig. S6E), consistent with the dynamic and cyclical nature of DZ-LZ transitions during GC responses. A more detailed analysis of key DZ- and LZ-associated genes revealed DZ-like subclusters that showed higher expression of *Cxcr4* and *Aicda* and that were *Bcl6^+^* (clusters 7 and 12 to 15), as well as clusters showing increased expression of markers consistent with LZ cells (*Cd83^+^*, *Cd86^+^*, or *Cd40^+^*; cluster 11) and putative preplasmablast cluster (*Bcl6^+^* and *Prdm1^+^*; cluster 9) (Fig. 4D). Further analysis of cell cycle gene expression within these GC subclusters also revealed dynamics consistent with genuine GC reactions (Fig. 4E). Specifically, DZ subcluster 12 showed an increased proportion of cells in G_1_ phase and high *Aicda* expression, aligning with the expected characteristics of DZ cells that are undergoing SHM (*25*, *26*). Moreover, other DZ-like clusters showed distinct cell cycle phases, with subcluster 7, 14, and 15 undergoing G_2_-M phase and subcluster 13 in S phase (Fig. 4E and fig. S6F). Hence, we concluded that the lung GCs represented genuine GC cells, with transcriptional patterns consistent with cells undergoing proliferation and SHM.

GC cells from cKO mice showed selective expansion of several DZ-like subclusters (clusters 7, 12, 14, and 15) (Fig. 4, A and B, and fig. S6D). These expanded clusters included those enriched for cells in G_1_ phase (clusters 12 and 15), a stage associated with SHM (*25*), and proliferating cells in G_2_-M phase (clusters 7 and 14) (Fig. 4E and fig. S6F). These data suggested that αv deficiency promotes expansion of proliferative and SHM-active B cells. We next asked what signals might drive the expansion of these DZ-like subclusters in the cKO mice. Given our previous work showing that αv down-regulates the strength of TLR signaling in B cells through action of autophagy proteins (*13*, *14*), we predicted that GC cells that are responsive to TLR signaling could be expanded in cKO mice. Supporting this, clusters 7, 12, 14, and 15 showed elevated expression of *Tlr*s compared with other clusters (Fig. 4F). Furthermore, lung GC B cells had higher expression of *Tlr*s than equivalent cells in the medLN, providing a potential explanation for the selective effects of αv deletion on lung B cells (Fig. 4G). Intriguingly, cluster 13, a DZ-like cluster that also showed higher expression of *Tlr*s, was only found in the medLN of the control mice. One of the highly expressed genes in this cluster was the autophagy gene *Map1lC3b*, and further analysis of GC-related autophagy genes, generated in our previous study (*14*), showed higher expression of two autophagy-related genes *Map1lC3b* and *Bnip3* in this cluster (fig. S6, G and H). We have previously shown both *Map1lC3b* and *Bnip3* to be involved in down-regulation of TLR signaling (*13*, *14*); therefore, higher expression of these autophagy related genes in cluster 13 likely suppresses TLR signaling despite elevated receptor expression, explaining why this cluster is not expanded in cKO lungs. These findings suggest that unchecked TLR signaling in αv-deficient B cells likely promotes selective expansion of TLR expressing DZ clusters in cKO mice.

To assess whether αv deficiency altered clonal dynamics, we also analyzed BCR sequences from GC B cells. Analysis of clonal diversity revealed that lung GC B cells from cKO mice had a lower inverse Simpson index than cells from controls, suggesting that cKO lung GC cells undergo greater clonal expansion (Fig. 4H). To further confirm this effect on clonal expansion, we identified expanded clones (defined as lineages with ≥3 cells sharing a common ancestor) in cKO and control mice. After controlling for differences in the total number of GC B cells in the two genotypes, cKO mice showed a visually dense network of shared clones in both lung and medLN, with visible increase in shared clones in the cKO lungs compared with those in the controls (Fig. 4I). Mapping clonal connections to GC subclusters showed that most of the expanded clones in lungs of cKO lungs were related to DZ clusters 7, 12, and 15 (Fig. 4J) but that expanded clones contributed cells to multiple clusters, linking the DZ subclusters with clonal expansion of GC cells in the cKO lungs.

Next, we asked whether the expansion of clones in cKO lungs resulted in increased diversification of antigen specificity. PR8-specific GC cells were present in both lung and medLN of all mice (Fig. 4K). However, cross-reactive GC cells (Cal09-HA^+^ or multiple positive: PR8-HA^+^/Cal09-HA^+^) were present at a much higher frequency in cKO mice than controls. PR8-HA^+^/Cal09-HA^+^ double-positive cells contributed around 5% of HA-specific cells in both lung and medLN of cKO mice but less than 1% of cells in control mice. This difference was even more pronounced for Cal09-HA^+^ single-positive cells, which made up almost 25% of GC B cells in cKO mice but were completely absent in lungs of control mice (Fig. 4, K to M). Comparative analysis of SHM in HA^+^ GC cells showed that both medLN and lung GC cells showed evidence of SHM across genotypes (Fig. 4N). While the overall mutation burden was similar between genotypes, the cKO lung GC cells exhibited a modest increase in the number of amino acid–replacement mutations per clone (Fig. 4O), suggesting more extensive antigen-driven diversification in the lungs of cKO mice.

Collectively, these results indicate that lung GC B cells represent canonical GC B cells and that αv integrin restrains the expansion of TLR-responsive DZ-like GC B cells in the lung. In the cKO mice, the loss of this regulation promotes clonal expansion and diversification of antigen specificity, leading to emergence of cross-reactive GC B cells, uniquely within the lungs.

### Loss of B cell αv induces distinct memory B cell subsets in the lungs

**Fig. 5.**
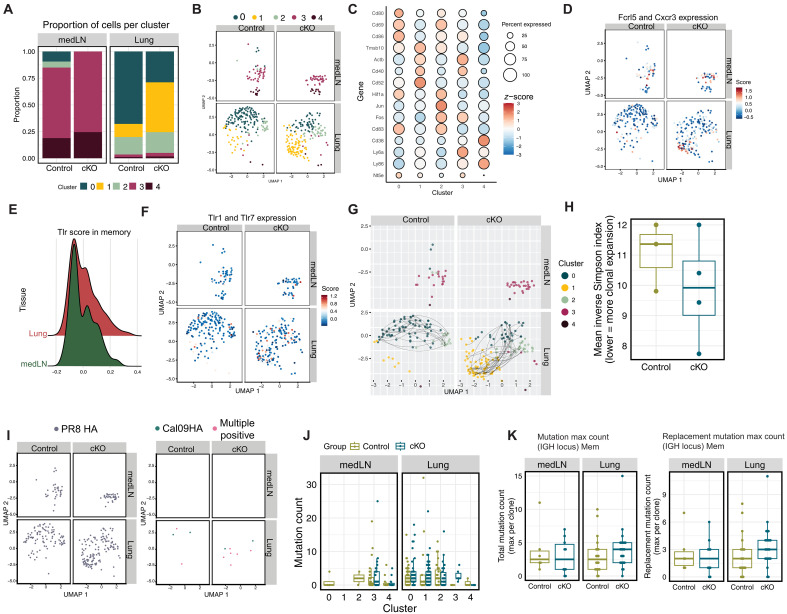
Loss of B cell αv induces distinct memory B cells in the lungs. scRNA-seq of sorted lung-resident or medLN PR8-HA^+^ or Cal09-HA^+^ B cells from control and cKO mice 20 days post–low-dose i.n. infection with live PR8. (**A** and **B**) Subclustering of the Cd80^+^ memory B cell cluster, cluster 6. (A) Quantification of the proportion of cells and (B) UMAP representation of the distribution per cluster in the medLN and lungs of the control and cKO groups. (**C**) Expression of marker genes within the different memory subclusters; color represents the expression level (*z*-score) of genes, and circle size represents the proportion of cells expressing that marker. (**D**) Expression of *Fcrl5* and *Cxcr3* on memory cells (calculated as in Fig. 4G) in medLN and lungs of control and cKO groups. (**E**) Distribution of TLR score (calculated as in Fig. 4G). (**F**) Expression score of *Tlr1* and *Tlr7* in the memory B cell subset. (**G**) Clonal sharing by BCR sequences, comparing clonality between memory cell subclusters in the medLN and lungs of the control and cKO group. Each dot represents a cell; each color represents a subcluster and each line connects a shared clone. (**H**) Clonal diversity of lung memory cells from control and cKO mice, quantified by inverse Simpson index; values are means of 100 replicates downsampled to a common number of lung memory cells per mouse. (**I**) UMAP representation of the PR8-HA–specific (left) and cross-reactive (Cal09-HA^+^ and multiple positive: PR8-HA^+^/Cal09-HA^+^) in the memory subclusters. (**J** and **K**) Quantification of the maximum number of total mutations by clone in memory subclusters (J) and in total memory (Mem) cells (K, left) and maximum number of replacement mutations in memory cells (K, right) in the medLN and lung of the control and cKO mice. Each dot represents one clone.

For analysis of memory B cells, we identified *Cd80*^+^ and *IgD*^−^ memory B cells as transcriptionally distinct from naïve B cells, while low expression of *Aicda* and *Prdm1* distinguished them from GC and plasma cells (fig. S6B). Expression patterns of *Bcl6*, *Cd38*, *Ccr6*, *Hhex*, and *Tle3* further confirmed the distinction of memory B cells from GC B cells (fig. S7, A and B). In contrast to GC B cells, which showed transcriptionally similar populations in lung and medLN, memory B cells showed distinct subclusters in the lung and medLN based on gene expression (Fig. 5, A and B; and fig. S7, C and D). In general, memory B cells from medLN displayed higher expression of genes associated with BCR signaling, antigen presentation, and apoptosis, whereas lung memory B cells were enriched for transcripts involved in cell-cycle progression, as well as nuclear factor κB (NF-κB), mitogen-activated protein kinase, and TLR signaling (fig. S7D).

cKO mice showed differences in the relative abundance of memory cell subclusters in the lung compared with controls (Fig. 5, A and B). Cluster 0, the predominant memory cell type in the lungs of control mice, expressed high levels of tissue-resident memory B cell markers *Cd69* and *Cd80* (Fig. 5C). In contrast, cluster 1, the predominant memory B cell population in lungs of cKO mice, expressed both canonical memory markers *Fcrl5* and *Cxcr3*, along with high expression of *Cd40* (Fig. 5, C and D). This cluster also exhibited elevated expression of genes involved in actin cytoskeleton remodeling *Actb* and *Tmsb10* (Fig. 5C), suggesting a distinct migratory or activation state. As with GC cells, lung memory B cells also showed increased expression of TLRs (Fig. 5E), and cluster 1 in particular had elevated expression of *Tlr7* and *Tlr1* (Fig. 5F). These data suggest that αv deficiency promotes expansion of distinct memory B cells in the lung.

We next investigated the clonal relationships among HA-specific lung and medLN memory B cells. Lung memory B cells showed substantial clonal expansion in both control and cKO mice, and single clones were represented across different memory subclusters (Fig. 5G), suggesting that these clusters represent different activation or differentiation states rather than distinct lineages. Lung memory cells from cKO mice also showed lower sequence diversity than controls, indicating increased clonal expansion (Fig. 5H), similar to our findings in GC B cells. In contrast, medLN memory B cells showed little to no clonal expansion in either genotype (Fig. 5G), suggesting little local proliferation of these cells. Most HA-specific lung memory B cells were specific for PR8, but a subset of cross-reactive (PR8/Cal09–double-positive or Cal09-specific) memory B cells was detected exclusively in the lungs (Fig. 5I). medLN-derived memory cells were exclusively PR8-specific and displayed lower levels of SHM compared with lung-derived counterparts (Fig. 5, I and J). Whereas small memory cell numbers limited statistical comparisons of SHM between genotypes, cKO lung memory cells showed higher total and amino acid replacement mutations (Fig. 5K). Overall, while mutation numbers trended higher, the increased clonal expansion of memory B cells in cKO lungs is consistent with their origin from highly active, prolonged GC responses.

Last, we used BCR sequences to determine the relationship between HA-specific cells in the medLN and lung and how this was affected by deletion of αv. Most of lung memory B cells shared sequences with medLN GC cells, consistent with initial activation and seeding from the lymph node (fig. S7, E and F). A subset of lung GC cells also shared sequences with medLN GCs. However, we also identified GC clones in the lung that did not share sequences with cells in the medLN, raising the possibility that some lung GC cells can develop independently of the medLN (fig. S7G). These data, together with the evidence of local clonal expansion (Figs. 4I and 5G), support a model in which, regardless of the tissue site where the clones originate, a subset of GC and memory B cells expands and evolves within the lung itself. This local evolution is enhanced in the absence of αv integrin, likely through increased responsiveness to innate cues such as TLR signaling, and promotes the emergence of mutated, cross-reactive, GC, and memory B cells at mucosal sites.

### Loss of B cell αv promotes sustained increase in lung-resident GC and memory B cells that are cross-reactive and of IgA isotype

**Fig. 6.**
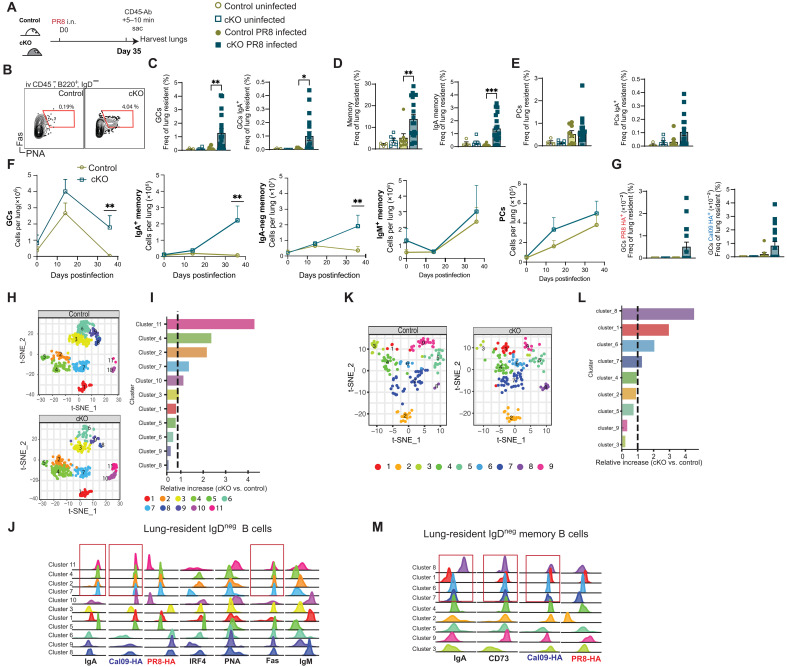
Absence of B cell αv sustains lung GC and memory B cells. Control and cKO mice were infected with low dose of live PR8 i.n. Thirty-five days later, all mice received r.o. injection of anti-CD45 antibody (α-CD45) 5 min before euthanasia. (**A**) Schematic for experimental procedure. Ab, antibody. (**B**) Representative flow plot of the GC cells; gated as resident (CD45-unlabeled) B220^+^ IgD^−^ IRF4^−^ Fas^+^ PNA^+^ from a control and cKO mouse. (**C** to **E**) Quantification of frequency of GCs and IgA^+^ GCs (C), Sw memory and IgA^+^ memory (D), and PCs and IgA^+^ PCs (E). Each dot represents one mouse; data are means ± SEM from combination of two experiments from three independent repeats. *P < 0.05, **P < 0.001, and ***P < 0.0001 by Mann-Whitney *U* test. (**F**) Line graph of number of lung-resident GCs, memory B cells, or PCs (plasma cells). Cell number was obtained as in Fig. 1. (**G**) Quantification of frequency of lung-resident GC cells specific for PR8-HA or Cal09-HA by flow cytometry. (**H**) t-SNE plot of HA-specific lung-resident IgD^−^ B cells [intravenous (iv) CD45^−^ B220^+^ IRF4^+/−^ IgD^−^ PR8-HA^+^/Cal09-HA^+^] from lungs with PhenoGraph clusters. (**I**) Fold increase of cell proportion per cluster in cKO versus control mice. (**J**) Histograms of expression of the markers included in the analysis for each cluster; red boxes highlight clusters with increased frequency in cKO mice. (**K**) Mice were infected i.n. with 100 plaque-forming units (PFU) of live PR8 IAV and rechallenged at day 31 with 50 PFU live PR8. Mice received r.o. injection with α-CD45 as above. t-SNE plot of lung-resident IgD^−^ memory B cells [intravenous (iv) CD45^−^ B220^+^ IRF4^+/−^ IgD^−^, Fas^−^ PNA^−^] with PhenoGraph clusters. (**L**) Fold increase in cell proportion per cluster in cKO versus control mice. (**M**) Histograms for expression of markers used for analysis in each cluster. Analysis is based on *n* = 6 mice per group in both sets of cluster analysis.

The expansion of lung-resident memory B cell subsets in the cKO mice suggested that these mice may exhibit stronger sustained anti-influenza B cell responses than controls. To test this, we measured lung-resident GC and memory B cell responses at day 35 post–PR8 IAV infection (Fig. 6A). By this time, lung-resident GC B cell frequencies and total numbers had returned to baseline levels in control mice but persisted in cKO mice, comprising up to 4% of IgD^−^ lung-resident B cells (Fig. 6, B and C). Notably, a small subset of the lung-resident GC B cells in cKO mice expressed IgA, which was not seen in controls (Fig. 6C). Similarly, lung-resident memory B cells persisted at significantly higher levels in cKO lungs compared with controls, with a marked increase in IgA^+^ memory B cells, whereas these had returned to baseline in controls (Fig. 6D). Plasma cell frequencies were low in both genotypes at this time point, although cKO mice showed a modest increase in IgA^+^ plasma cells (Fig. 6E). Analysis of these subpopulations over time showed a specific increase in lung-resident GC and memory B cells in the cKO mice. Moreover, while IgA^+^ memory B cells comprised a small portion of lung-resident memory B cells, we observed a significant increase in this population over time in the cKO lungs while there was a minimal increase in this population in the control lungs around day 14. In contrast, there were no significant increases in IgM^+^ memory B cells or total plasma cells in the cKO lungs over time indicating specific effects of αv deletion on sustained increase in GC and memory B cells (Fig. 6F).

We next evaluated the antigen specificity of these lung-resident B cells using the same HA tetramers that we used for RNA-seq studies (fig. S8A). PR8-HA–specific and Cal09-HA–reactive GC B cells were still detectable in cKO mice at day 35 but absent in control mice (Fig. 6G). Antigen-specific memory B cells were rare overall, with PR8-specific memory cells found only in cKO lungs (fig. S8B) while frequencies of Cal09-reactive memory were very low in both genotypes (fig. S8C).

To further characterize these small numbers of antigen-specific cells that were expanded in the cKO mice, we performed high-dimensional clustering analysis of lung-resident IgD^−^ HA-specific B cells using PhenoGraph (Cytofkit, Bioconductor). This analysis identified 11 statistically distinct clusters (Fig. 6H). We found that four of these clusters (11, 4, 2, and 7) had a higher frequency of cells in the cKO mice, compared with those in controls, and we assessed the characteristics of these expanded cells (Fig. 6I). All of these four clusters showed higher IgA expression and increased staining for Cal09-HA, compared with other clusters (Fig. 6J). Moreover, these four clusters also showed increased expression of GC-associated markers (Fas or PNA), compared with other clusters (Fig. 6J). In contrast, the clusters that had a higher frequency in the control mice compared with the cKO (cluster 6, 9, and 8) showed increased PR8-HA staining and IRF4 expression (Fig. 6J), indicating increase in plasma cells recognizing infecting virus. This analysis further confirmed that the loss of αv promotes the persistence of IgA-expressing, cross-reactive GC B cells in the cKO lung.

To confirm the antigen reactivity of the memory B cells that were expanded in the cKO lungs, we performed a similar cluster analysis on memory B cells generated at day 4 post-rechallenge, when recall response would be at peak and provide amplification of memory B cells for analysis. We also used CD73 as a positive marker for memory B cells, based on our scRNA-seq analysis, where we found expression of CD73 (Nt5e) on memory subclusters in the lung. We performed PhenoGraph cluster analysis within the lung-resident IgD^−^ memory B cells (Fig. 6K) and identified four clusters (8, 1, 6, and *7*) that were more frequent in cKO mice (Fig. 6L). These four clusters were highly positive for Cal09-HA and the marker CD73 (Fig. 6M). There was only one cluster showing high IgA expression, cluster 8 (Fig. 6M). This was one of the clusters with increased frequency of cells in the cKO mice and showed high CD73 expression as well as increased staining for Cal09-HA (Fig. 6M). CD73 is a marker associated with GC-derived memory B cells (*27*); therefore, these findings indicate that the loss of B cell αv leads to sustained increase in GC B cells in the lungs after infection, which leads to increases in cross-reactive IgA^+^ memory B cells.

### Persistence of lung GC activity in the B cell αv integrin-deficient mice enhances cross-reactive mucosal immunity

To confirm that the observed increases in lung-resident IgA^+^ GC and memory B cells in cKO mice are due to ongoing GC activity in iBALTs, we analyzed lungs of cKO and control mice at day 36 postinfection by confocal microscopy.

**Fig. 7.**
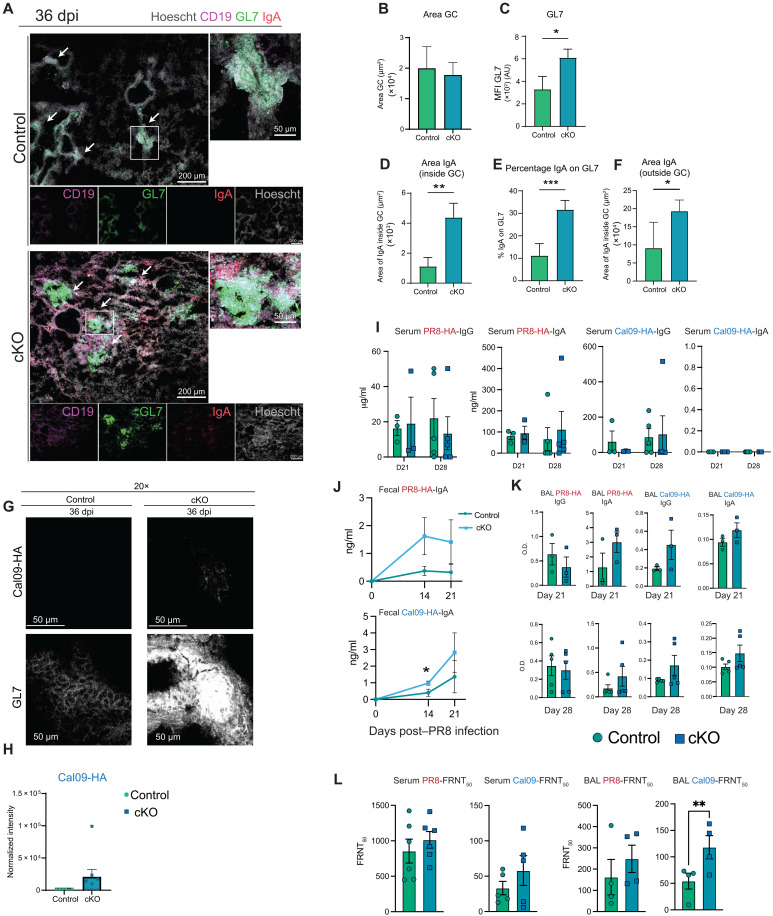
Absence of B cell αv leads to persistent lung GC activity. Control and cKO mice were infected with low dose of live PR8 i.n. Lungs were harvested 36 days postinfection (dpi). (**A**) Representative confocal images of lung sections of one single plane at 20× magnification. Scale bars, 200 μm. Hoechst (gray), CD19 (magenta), GL7 (green), and IgA (red). Arrows show GL7^+^ GCs. Insets highlight GCs. Scale bars, 50 μm. (**B** to **F**) Bar graph showing means ± SEM of GC area in μm^2^ (B), GL7 mean fluorescence intensity (MFI) within GCs (C), IgA area inside GC (D), percentage of IgA staining overlapping with GL7 within GC (E), and IgA area outside GC (F). Quantifications from 9 to 12 different fields of sections at 10×, three mice per genotype (*n* = 12 to 27). (**G**) Representative confocal images of lung iBALT-GCs, stained for Cal09-HA and GL7 at 20×. (**H**) Bar graph of normalized intensity of Cal09-HA staining from six to eight fields of sections viewed at 20× from three mice per genotype; *P < 0.05, **P < 0.01, and ***P < 0.001 by Mann-Whitney *U* test. (**I** to **K**) Serum, BAL fluid, or feces were isolated form mice infected with low-dose PR8 for quantification of PR8-HA– or Cal09-HA–reactive antibodies by enzyme-linked immunosorbent assay (ELISA). Concentrations for indicated antibodies or optical density values are presented as bar or line graphs. Line graphs show means ± SEM with *n* = 4 to 5 mice per group for fecal samples; *P < 0.05, Mann-Whitney test comparing control and cKO groups at each time point. Bar graphs show individual mice as dots with means ± SEM; *n* = 3 mice per group for day 21 and *n* = 5 mice per group for day 28. (**L**) Neutralizing capacity of antibodies in serum or BAL at 28 days postinfection, against H1N1 PR8 or H1N1 Cal09 virus, shown as FRNT_50_, with *n* = 5 to 6 mice per group for serum and *n* = 4 mice per group for BAL. Graphs show individual mice as dots with means ± SEM. **P < 0.01, paired *t* test.

At day 36 postinfection, cKO mice had identifiable GC clusters in iBALT areas, consistent with the detection of GC B cells by fluorescence-activated cell sorting (FACS). Curiously, we also detected areas of GL7-staining cells in control mice, and these were of a similar size to those in cKO mice (Fig. 7A). This was at odds with our FACS analysis, in which we detected fewer GC B cells in control mice. However, this apparent discrepancy may be due to the much lower staining for GL7 in the control mice (Fig. 7, A to C). Notably, cKO mice showed a large increase in IgA^+^ cells in the lung compared with controls (Fig. 7A). Quantification confirmed that both the total area of IgA^+^ cells (Fig. 7D) and the percentage of IgA^+^ GL7^+^ (Fig. 7E), within iBALT-GC structures, remained significantly elevated in cKO mice. Moreover, in the cKO lungs, we saw significant amount of IgA^+^ cells also outside the GC areas that we interpret as the lung-resident IgA^+^ memory B cells (Fig. 7F) observed in the flow cytometry analysis. We also observed Cal09-HA^+^ cells within GC regions of cKO lungs, but these were not detected in controls (Fig. 7, G and H), further supporting the presence of cross-reactive GC B cells locally in the lungs of the cKO mice.

To assess whether these sustained lung GC reactions in the cKO mice affect mucosal immunity, we compared influenza-specific and cross-reactive antibodies in serum, feces, and bronchoalveolar lavage (BAL) fluid from control and cKO mice following PR8 infection. In serum, PR8-reactive IgG antibodies were induced in both genotypes, with no differences between groups (Fig. 7I). Cal09-reactive IgG antibodies not only were found in lower levels than the PR8-specific antibodies but also did not differ between genotypes. Serum IgA responses to either PR8 or Cal09 were markedly lower than IgG responses in both groups, consistent with the mucosal restriction of IgA after intranasal infection and showed no genotype-dependent differences (Fig. 7I).

We next examined mucosal compartments for further analysis of mucosal antibodies. Because respiratory mucosal infection can induce antigen-specific IgA detectable in distal mucosal secretions, including feces (*28*, *29*), we quantified fecal IgA antibodies before infection and up to day 21 postinfection. cKO mice consistently exhibited increased PR8-specific IgA and, notably, higher levels of cross-reactive Cal09-specific IgA compared with controls (Fig. 7J), representing the most reproducible enhancement of antibody responses observed across compartments. To directly assess airway-associated antibody responses, we also analyzed BAL fluid at days 21 and 28 postinfection. PR8-specific IgG levels were comparable between genotypes at both time points, whereas PR8-specific IgA levels showed trend toward increase in the cKO mice (Fig. 7K). Cal09-reactive IgG and IgA were detected at low levels and exhibited variability, with trends toward higher responses in a subset of cKO mice (Fig. 7K). Thus, BAL antibody responses were variable and showed a trend toward increase in cross-reactive mucosal antibodies in the cKO mice although this did not reach statistical significance.

Last, to assess functional activity, we performed neutralization assays using serum or BAL isolated at day 28 postinfection. Serum neutralization titers against PR8 were similar between genotypes. While Cal09 neutralization did not differ significantly at the group level, several cKO mice exhibited higher Cal09-neutralizing titers compared with controls (Fig. 7L). In contrast, BAL fluid from cKO mice showed significantly increased neutralizing activity against Cal09, whereas PR8 neutralization in BAL was not significantly different. Together, these data show that αv deficiency augments lung-localized GC-dependent immunity, most consistently reflected by significantly increased fecal IgA and enhanced cross-reactive neutralizing activity in the lungs.

### B cell αv integrin regulates IgA differentiation in a B cell–intrinsic manner

Our scRNA-seq analysis revealed that the expansion of GC B cells in the lungs of αv-deficient (cKO) mice was associated with elevated expression of TLRs, particularly within DZ-like subclusters. Consistent with this, we previously showed that αv integrin restrains TLR signaling in systemic immune responses, limiting TLR-induced class switching to IgG2c. However, it is unclear whether increased TLR signaling could be responsible for the expansion of IgA–class-switched cells that we see in lungs of cKO mice. To test this, we used in vitro models of B cell differentiation and class switching.

**Fig. 8.**
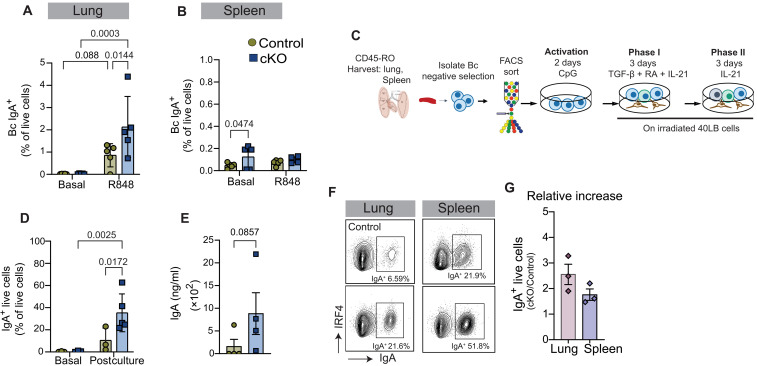
TLR-ligand stimulation leads to the differentiation and proliferation of tissue resident IgA–class-switched B cells. (**A** and **B**) Lung and spleen B cells were isolated from uninfected control (C57B6) and cKO mice and cultured in a 96 well plate with R848 (1 μg/ml) for 4 days. IgA expression was assessed by flow cytometry. Quantification of the frequency of IgA-expressing B cells in the lungs (A) and spleen (B) of control and cKO mice. (**C**) Schematic of the experimental procedure. C57BL/6 (control) and αv^fl/fl^ CD19^Cre+^ (cKO) mice were injected r.o. with 1 μg of α-CD45-APC 5 min before euthanasia. Lung and spleen were then collected and sorted for intravenous CD45-unlabeled resident cells and plated for a total of 8 days. Cells were activated with CpG (1 μg/ml) for 2 days and then added TGF-β (2 ng/ml), retinoic acid (RA; 100 nM) and interleukin-21 (IL-21; 40 ng/ml). Bc, B cells. (**D**) Frequency of IgA-expressing live cells within lung-resident B cells after culture as determined by flow cytometry. (**E**) Secreted IgA as measured by ELISA of the supernatant of lung-resident B cells after TGF-β, RA, and IL-21 culture. (**F**) Representative flow plots of lung and spleen resident B cells after the entire 8-day culture protocol. Cells were gated from live lymphocytes. (**G**) Relative increase in the frequency of IgA^+^ live cells, in the cKO versus the control cultures comparing cultures from lung and spleen B cells after the 8-day culture protocol. Each dot represents culture with cells from individual mice from three to five independent experiments. Bar graphs show means ± SEM; statistical significance was determined by two-way ANOVA (A, B, and D) or Mann-Whitney *U* test (E).

We first tested whether TLR stimulation could promote greater amounts of IgA class switching in the absence of αv. B cells isolated from either lung or spleen were stimulated with the R848, a synthetic ligand for TLR7, and IgA class switching was measured by flow cytometry. R848 was sufficient to promote generation of IgA^+^ B cells in the lung B cell culture, and approximately twice as many IgA^+^ B cells were generated from lungs of cKO mice than controls (Fig. 8A), in agreement with our model. In contrast, B cells from the spleen, regardless of the genotype, did not differentiate into IgA cells under these conditions (Fig. 8B). We speculated that this may reflect the lack of exposure of spleen B cells to the mucosal conditioning signals such as retinoic acid (RA) and transforming growth factor–β (TGF-β) that promote IgA class switching. To more comprehensively model the full process of IgA class switching and assess the contribution of TLR signaling, we turned to a multistep culture protocol that better models all aspects of B cell activation and IgA differentiation (*28*, *29*). Total IgA^−^ lung-resident B cells from lung were first activated via TLRs using CpG (a TLR9 ligand) to engage both naïve and activated B cells, followed by costimulation with B-cell activating factor (BAFF) and CD40L to mimic GC-like conditions. Interleukin-21 (IL-21), RA, and TGF-β were also included to promote IgA class switching (Fig. 8C). Under these conditions, IgA^+^ B cells were generated in all cultures, at much higher frequency than in cells treated with R848 alone. However, cKO B cells consistently generated higher proportions of IgA^+^ B cells compared with control B cells (Fig. 8D). In parallel, significantly more IgA was detected in the supernatants of cell cultures from cKO mice (Fig. 8E). Cultures of B cells isolated from spleen also led to class switch to IgA isotype (Fig. 8F), and the spleen B cell culture from cKO mice showed increase in IgA^+^ cells. However, the fold increase in IgA^+^ cells was significantly greater in lung-derived cKO B cells than in spleen-derived cells (Fig. 8G), suggesting that mucosal imprinting enhances TLR-driven IgA differentiation.

Together, these findings demonstrate that B cell–intrinsic αv integrin limits TLR-mediated class switching to IgA, particularly in mucosally conditioned lung B cells. Loss of αv enhances the responsiveness of these cells to TLR signals, resulting in increased IgA class switching in vitro*.* This is consistent with our in vivo findings of sustained increase in IgA^+^ GC and memory B cell responses in the lungs of cKO mice.

## DISCUSSION

Recent studies have shown that natural infection of the respiratory tract by influenza virus induces memory B cells that are lung resident (*2*), major producers of broadly reactive antibodies (*30*) and IgA isotype antibodies (*2*, *4*). These tissue-resident B cells contribute substantially to secondary protection but are not efficiently generated by systemic vaccines, highlighting a critical need to understand the local cues that shape mucosal B cell immunity. Here, we identify B cell αv integrin as a key negative regulator of GC persistence, clonal evolution, and IgA^+^ memory B cell development in the lung following influenza virus infection. Loss of B cell αv promoted expansion of GC and memory B cells in the lung and generation of cross-reactive B cells in the lungs compared with those in the medLN, revealing a spatially restricted mechanism of mucosal B cell regulation.

GCs are sites of affinity maturation of B cells, where B cells undergo successive rounds of proliferation, SHM, and selection, through antigen presentation to T follicular helper cells (*31*). Studies on influenza and other viral infections have revealed that increasing SHM in the GCs increases not only the affinity of antibodies for immunizing antigens but also their breadth, enabling recognition of diverse viral strains (*32*, *33*). While we previously showed that αv loss enhances GC responses and SHM in secondary lymphoid organs, its role in mucosal B cell responses was unknown. Here, we show that αv-deficient B cells form persistent, ectopic GCs within iBALT in the lungs (Fig. 7), leading to expanded clones with elevated SHM and increased cross-reactivity to heterologous HA variants (Figs. 4 and 5). These GC structures persist for at least 35 days postinfection and support the formation of IgA^+^ memory B cells in the respiratory mucosa. Although lung iBALT-GCs are strongly associated with cross-reactive memory, our previous work on spleen GCs indicates that αv deficiency can enhance the breadth of systemic GC outputs as well. Thus, the increased cross-reactive cells in cKO mice likely reflect a global increase in GC activity, with lung iBALT contributing substantially due to its persistence. Future studies disrupting lung GCs would be important to distinguish the contributions of lung GCs versus medLN GCs.

Using single-cell transcriptomics of antigen-specific B cells, we observed that influenza infection induces GC and memory B cells with shared clonal origins in the lung and medLN. Although comparisons between lung and lymphoid tissues can be influenced by differences in tissue processing, lung leukocytes require enzymatic digestion, whereas medLN cells can be isolated mechanically, the major GC subclusters were recovered robustly from both tissues and displayed consistent canonical marker expression and internal transcriptional architecture. Moreover, control and αv-deficient lung samples were processed identically, ensuring that the genotype-dependent differences that we observed cannot be attributed to digestion-related artifacts. Beyond validating our flow cytometry findings of increased Cal09-binding and cross-reactive B cells in αv-deficient lungs, the scRNA-seq data revealed several unanticipated features of the response: B cell αv deficiency selectively expanded TLR-responsive GC and memory subclusters in the lung. Moreover, whereas GC B cells exhibited strong transcriptional similarity between lung and medLN, memory B cells were transcriptionally distinct across the two compartments (Figs. 4 and 5). These findings underscore the tissue-specific nature of αv-mediated regulation. Lung-resident memory B cells in these mice also exhibited greater clonal expansion and broader HA specificity than their medLN counterparts (Fig. 5), indicating that prolonged GC activity at mucosal sites shapes the local memory B cell pool. These findings align with previous studies showing that lung-resident memory B cells are key contributors to cross-reactivity (*30*) and underscore that local B cell evolution driven by tissue-intrinsic cues, likely involving enhanced innate immune signaling are key to promoting expansion of cross-reactive B cells in the lungs.

We have previously shown that the loss of B cell αv enhances responses from innate-like B cell populations such as B1 B cells, marginal zone B cells, as well as extrafollicular B cells (*6*, *15*). These B cell populations can all be involved in the immune response against respiratory viruses (*1*, *34*) and likely lead to IgA production at the respiratory tract associated with early clearance of the virus. But, here, we focused on lung-resident GC-derived B cells that emerge later and are more relevant for secondary protection. In αv cKO mice, persistent iBALT structures supported accumulation of IgA^+^ GC and memory B cells from day 16 through day 36 postinfection (Figs. 3 and 7). Upon secondary challenge, cKO lungs harbored increased numbers of CD73^+^ IgA^+^ and Cal09-binding memory B cells (Fig. 6). While we cannot rule out the possibility that these memory B cells could be generated from GC reactions in the medLN, based on our RNA-seq data, we predict that the increases in cross-reactive IgA memory B cells in the lungs of cKO mice are, at least, in part, due to increased expansion of GC and memory B cells in the lungs.

Although IgA^+^ cells were a minor fraction of the lung-resident memory B cell pool, their selective enrichment in the cKO mice underscores a previously unrecognized opportunity to drive GC-derived long-lived mucosal IgA immunity. While IgA^+^ cells were underrepresented in our scRNA-seq dataset, likely due to low abundance, transcriptomic analysis revealed distinct lung memory B cell subsets enriched for TLR, NF-κB, and cytoskeletal remodeling pathways-signatures associated with heightened activation and tissue residency. We predict that these transcriptional programs are linked to the expanded IgA^+^ memory population in cKO lungs and arise via TLR-driven signals. Further studies at later time points will be necessary to confirm this relationship. These scRNA-seq data provide transcriptional signatures that distinguish lung and medLN memory subsets, which will enable functional dissection of the protective capacity of lung-resident memory cells in future. Moreover, while further studies are still necessary to establish the full heterosubtypic protective capacity of lung-resident GC and memory responses against multiple influenza strains, our studies provide a basis to design these studies.

Mechanistically, our in vitro experiments demonstrated that B cell–intrinsic loss of αv enhanced IgA class switching in response to TLR stimulation, especially under conditions mimicking mucosal cytokine environments (Fig. 8). This aligns with prior findings that αv deficiency leads to enhanced TLR-induced expression of *Aicda* (*14*, *16*), a critical gene for class-switch recombination and SHM. Notably, the increase in IgA class switching was more pronounced in B cells derived from the lung than from the spleen, further supporting a tissue-specific effect.

Although loss of αv also enhances IgG responses, we focused here on IgA^+^ B cells because their regulation within respiratory tissues remains poorly defined. While TGF-β–driven IgA class switching and preferential selection of IgA^+^ B cells have been characterized in gut Peyer’s patch GCs (*35*), the mechanisms governing IgA differentiation in the lung are far less understood. Our combined analyses of IgG^+^ and IgA^+^ GC B cells in the lung, together with IgG and IgA antibody measurements in BAL fluid, feces, and serum, indicate that loss of αv-driven TLR signaling broadly amplifies mucosal B cell responses, with particularly pronounced effects on IgA in lung. As studies have demonstrated that IgG^+^ B cells can undergo class switching to IgA in the gut (*36*), we speculate that prolonged TLR signaling and enhanced GC persistence resulting from αv loss may promote both expansion and secondary isotype switching within lung GCs, thereby favoring the accumulation of IgA^+^ GC and memory B cells observed in cKO mice. Together with our prior work, our current data suggest that αv integrins restrain mucosal B cell responses through regulation of TLR signaling and that prolonged TLR signaling due to loss of αv enhances B cell proliferation and CSR to drive the amplified IgA^+^ GC and memory responses in the lung.

Our findings highlight B cell αv integrin as a checkpoint that constrains mucosal GC activity and IgA^+^ memory B cell diversification in the respiratory tract. Given growing evidence that mucosal IgA responses limit infection and transmission of respiratory viruses more effectively than systemic IgG responses (*4*, *37*, *38*, *39*), these findings offer a promising strategy for improving respiratory virus vaccines. A key challenge with current respiratory viral vaccines is the lack of protection from infection at respiratory sites as well as the lack of durability of protection induced by the vaccines. Strategies that prolong GC activity in the lung through intranasal vaccines could both drive protection at respiratory sites and promote long-lived GC-driven IgA^+^ memory B cell responses. Intranasal vaccines incorporating αv blockade or TLR ligand adjuvants may be particularly effective in this regard. Future studies on pharmacological αv integrin blockade and its overall consequences would be valuable to assess the translation capacity of these findings. Moreover, studies on the effects of TLR ligand adjuvants in mucosal vaccines could similarly be used to specifically promote lung-resident long-lived B cell responses for vaccine efficacy.

## MATERIALS AND METHODS

### Mice

αv^fl/fl^ mice were backcrossed to C57BL/6J mice as previously described (*13*, *16*). The animals were bred and maintained on the C57BL/6 background. αv^fl/fl^ mice were crossed with CD19 cre transgenic mice to generate B cell–specific αv knockout mice, referred to as cKO mice (CD19^cre/+^ αv^fl/fl^). Mice with a single CD19^Cre^ allele, CD19^cre/+^αv ^+/+^, were used as controls. For the in vitro experiments in Fig. 8, C57BL/6 mice were used as controls. All mice for this study were age and sex matched and were 9 to 14 weeks old at the time of infection. Mice were housed in a specific pathogen–free facility. All procedures were approved by the Institutional Animal Care and Use Committee at Seattle Children’s Research Institute (protocol number IACUC00580).

### Influenza infection

Live PR8 IAV was purchased from AVS Bio (Charles River Laboratories Avian Vaccine Services) and stored in single-use aliquots at −80°C. Just before infection, PR8 was thawed and diluted in phosphate-buffered saline (PBS). Mice were anesthetized with Isoflurane (Patterson Veterinary) and infected with PR8 IAV in 25 μl in the nostrils with 100 to 200 plaque-forming units of live PR8 IAV for low-dose infection studies. This low dose was chosen as the dose that led to some weight loss but did not lead to significant weight loss or mortality after infection. Mice were weighed and monitored for signs and symptoms of disease for the duration of the study. Live H1N1 Cal09 virus was purchased from American Type Culture Collection (ATCC) and used for neutralization studies.

### HA tetramer preparation

The B cell HA tetramers were gifts either from E. Gage (*17*) or from K. Masaru’s lab at National Institutes of Health (NIH). Tetramers with an AviTag were biotinylated with the BirA biotin-ligase kit (Avidity) according to the manufacturer’s instructions and stored in single-use aliquots at −80°C. Following biotinylation, BV711 (BioLegend)– or PerCpCy5.5 (BioLegend)–conjugated streptavidin (SA) or TotalSeq-SA-C0951-PE (BioLegend) was added in five serial steps with a 10-min incubation period each to the unlabeled tetramers. Labeled tetramers were prepared the day before the experiment.

### Antibodies

α-IgD-BV786 (563618), α-B220-BUV395 (563793), α-CD45-BUV395 (564279), and α-CD19-BUV737 (612781) were purchased from BD Biosciences. Α-CD3-BV510 (100234), α-CD11c-BV510 (117337), α-F4/80-BV510 (123135), α-CD138-BV421 (142523), α-Fas-BV605 (152612), SA-PerCPCy5.5 (405214), SA-BV711 (405241), α-IgM-PerCPCy5.5 (406512), and TotalSeq-C0301-C0315 and TotalSeq-SA-C0951 (for scRNA-seq) were from BioLegend. α-CD45-APC (allophycocyanin) (17-0451-82), α-IRF4-PeCy7 (25-9858-82), and α-IRF4-AF700 (56-9858-82) were purchased from Invitrogen. α-IgA-Biotin (1040-08), α-IgA-PE (1040-09), and α-IgM-PeCy7 (1140-17) were from SouthernBiotech. α-Gr1-BV510 (130-120-824) and PNA–fluorescein isothiocyanate (FITC; FL-1071) were from Miltenyi Biotec and Vector Labs, respectively.

### Flow cytometry

Five minutes before euthanasia, mice were injected with 1 μg of α-CD45-BUV395 or α-CD45-APC in the retro-orbital cavity. Lungs and medLNs were collected from euthanized mice. Lung tissue was digested with collagenase (1.5 mg/ml; MiliporeSigma) and deoxyribonuclease I (10 μg/ml; MiliporeSigma) in Mg^2+^, Ca^2+^-free Hanks’ balanced salt solution (Gibco) with 10% fetal bovine serum (FBS; Sigma-Aldrich) for 1 hour at 37°C or 30 min in a shaking incubator at 1000 rpm. Lungs and medLN were then ground into a 40-μm filter to generate a single-cell suspension. For some experiments, lung B cells were isolated using CD19-positive magnetic bead separation (STEMCELL Biotechnologies). Cells were counted using AccuCheck count beads (Thermo Fisher Scientific) or Muse Cell Analyzer (Luminex) according to the manufacturer’s instructions. Cells were then stained with fixable LiveDead Aqua (Thermo Fisher Scientific) to identify dead cells, after which cells were stained with PR8-HA and Cal09-HA B cell tetramers and surface antibodies for 20 min at room temperature. To identify intracellular antigens, cells were then fixed and permeabilized with the Transcription Factor kit (BioLegend) according to the manufacturer’s instructions. Events were analyzed on an LSR II flow cytometer (BD Biosciences) or an LSRFortessa (BD Biosciences) and analyzed using FlowJo (v.10, TreeStar). See fig. S1 for gating strategies.

Cells for sorting were prepared as a single-cell suspension (as described above) from lungs and medLN of PR8 IAV infected mice. Five minutes before euthanasia, mice were injected with 1 μg of α-CD45-APC in the retro-orbital cavity. Before staining, cells were blocked with Fc block for 10 min and then stained with fixable LiveDead Aqua (Thermo Fisher Scientific) to identify dead cells, surface antibodies, hashtag oligos for each mouse, and PR8-HA and Cal09-HA B cell tetramers for 20 min at 4°C. Cal09-HA and PR8-HA tetramers were bound to SA-Oligo–tagged PE (phycoerythrin) and SA-BV711 to gate out SA-PE–specific B cells from the lungs and medLN. All cells from each mouse were combined into a single pool per tissue and sorted to enrich for tetramer-specific cells gated as live CD45-unlabeled B220^+^ HA tetramer^++^.

### ELISpot

ELISpots were performed on single-cell suspensions of lung or medLN cells, prepared as described above. MAIPS (MultiScreen-AIP Sterile) ELISpot plates (Merck Millipore) were precoated with PR8-HA or Cal09-HA (3 μg/ml; SinoBiological) diluted in PBS. Serial dilutions of cells were plated over wells and incubated overnight at 37°C. Spots were developed using α-mouse IgG or α-mouse IgA alkaline phosphatase (AP) Abs (SouthernBiotech) followed by 5-bromo-4-chloro-3-indolyl phosphate/nitro blue tetrazolium (BCIP/NBT) AP substrate (Vectors Labs). Spots were imaged and counted using an ImmunoSpot analyzer (Cellular Technology Limited).

### Histology and immunofluorescence

Lungs were perfused with PBS and then inflated and embedded in optimal cutting temperature compound (OCT) and frozen at −80°C [as described in (*40*)]. Frozen sections (8 μm) were fixed in acetone at −20°C, air-dried, and blocked with 1% normal goat serum (Jackson ImmunoResearch) and 1% bovine serum albumin (BSA; Sigma-Aldrich) in PBS with 0.1% Tween 20 (Sigma-Aldrich). Slides were stained with Hoechst, α-CD19-AF647, α-GL7-FITC, and α-IgA-PE. Images were acquired with a Zeiss LSM 900 confocal microscope. Image processing and analysis were performed with Fiji (ImageJ) software (*41*), with a personalized macro where the iBALT structure was manually delimited by the polygon tool, and, then, the image was converted to a mask by default threshold to measure the area of IgA- and GL7-positive staining by the “analyze particle” function. IgA^+^GL7^+^-positive area percentage was calculated by applying the image Calculator “AND” function over IgA and GL7 masks, then measuring the resulting area by analyze particle function, and calculating the percentage of the resulting area over the whole GL7-positive area.

### Single-cell RNA sequencing

B cells were isolated from lung and medLN from mice at 20 days postinfection with PR8 influenza (as described above). Enriched HA tetramer–specific B cells were run on 10x Chromium with 5′ GEM-X kit to isolate single cells, and libraries were generated for gene expression, BCR sequencing, and hashtag oligo quantification. Libraries were sequenced on an Illumina NextSeq 2000 following 10x guidelines for read formats. Libraries were processed to gene and hashtag unique molecular identifier (UMI) counts and assembled BCR sequences with isotype, using cellranger v. 8.0.0. Downstream analysis was performed using Seurat v. 5.1 (*42*). Cells were filtered for quality using standard metrics, expression of target, and nontarget cell type genes and then demultiplexed using hashtag oligo counts. Cells were further filtered on the basis of detection of at most one heavy and two light BCR chains, yielding 9660 high-quality single B cells. Normalization, Uniform Manifold Approximation and Projection (UMAP), clustering, and marker gene identification were performed with Seurat, and clusters were assigned to major B cell subsets using marker genes *Ighd*, *Aicda*, *Cd80*, and *Prdm1*. Cells from mice lacking substantial GC B cell populations in the medLN were excluded from downstream analysis. Cells were called positive or negative for each HA tetramer using a threshold on log-transformed barcode UMI counts. We used SCENIC to identify modules of genes putatively regulated by transcription factors (*24*).

Analysis of BCR sequences was conducted using the Immcantation toolkit. We used Change-O to assign sequences to V and J genes, define clones by matched using heavy- and light-chain genes and an empirical threshold on CDR3 Hamming distances, and infer germline sequences (*43*). Trees were inferred using the dowser implementation of IgPhyML and the HLP19 model on paired heavy and light chains (*44*–*46*). SHM was quantified using SHazaM (*43*). Clonal diversity metrics were calculated by repeatedly downsampling to a common number of cells per mouse in the focal population and then calculating the metric on each downsampled replicate.

### In vitro plasma cell culture

All mice were injected retro orbitally with 1 μg of α-CD45-BUV395 5 min before euthanasia. B cells from spleen, medLN, or lung were isolated using negative magnetic bead separation (STEMCELL Biotechnologies). Cultures with R848 only were performed on Lympholyte-M (Cedarlane) separated lung B cells and B cells from spleen isolated using negative magnetic bead separation. B220^+^ B cells were then sorted for intravenous CD45^−^ populations using an FACSAria sorter (BD Biosciences). Plasma cells were differentiated from naïve B cells. Briefly, cells were suspended in Iscove’s modified Dulbecco’s medium (Cytiva) supplemented with 10% FBS (Sigma-Aldrich), 2 mM GlutaMAX (Gibco), 55 μM β-mercaptoethanol (Gibco), penicillin (100 U/ml; Gibco), and streptomycin (100 μg/ml; Gibco) and activated with CpG (1 μg/ml; InvivoGen) for 2 days. Cells were then seeded over irradiated 40LB feeder cells, a gift from D. Kitamura (*29*), expressing mouse CD40L and BAFF. To induce class switch to IgA, cells were incubated with TGF-β (2 ng/ml; BioLegend), 100 nM RA (Sigma-Aldrich), and IL-21 (40 ng/ml; PeproTech) for 3 days, followed by 3 days in IL-21 alone.

### ELISA and virus neutralization assay

Enzyme-linked immunosorbent assays (ELISAs) were performed on the supernatant of cultured cells (see the “In vitro plasma cell culture” section for culture details). Immulon 2HB plates (Thermo Fisher Scientific) were precoated with PR8-HA or Cal09-HA (1 μg/ml; Sino Biological) diluted in PBS. Serial dilutions of supernatant were plated and left overnight at 4°C. The plates were developed using α-mouse IgA or IgG-AP antibodies (SouthernBiotech) followed by 4-nitropenyl phosphate disodium salt hexahydrate substrate tablets (Sigma-Aldrich). Absorbance (optical density) was measured using a SpectraMax 190 luminometer (Molecular Devices) at 405 nm. For neutralization assays, serum or BAL samples were receptor-destroying enzyme (RDE treated, serially diluted in minimum essential medium, mixed with virus (H1N1 CAL09 or H1N1 PR8), and incubated for 1 hour at 37°C. Dilutions were then added to Madin-Darby canine kidney (MDCK) cell monolayers, and cells were infected for 1 hour, overlaid with methyl cellulose, and incubated for 24 hours in the presence of trypsin. Cells were then fixed with formaldehyde, permeabilized with methanol, blocked with BSA, and stained using detection monoclonal antibody (19C10-biotin) system followed by SA-AP and BCIP/NBT substrate to visualize foci, as adapted from the FociSpot Mabtech protocol (kit reference 4405-4AT). Foci were quantified with a Mabtech IRIS reader, and neutralization titers were reported as 50% Focus Reduction Neutralization Test (FRNT_50_) values, defined as serum or BAL dilution causing a 50% reduction in foci relative to virus-only controls. MDCK cells were purchased from ATCC (MDCK cell line; PTA-6500).

### Statistical analysis

Raw data from control and cKO PR8-infected groups were analyzed by Mann-Whiney *U* test, two-way analysis of variance (ANOVA), or paired *t* test, using GraphPad Prism (v.9.3.1). *P* < 0.05 was considered statistically significant.
