# Supplementary material for: B cell αv integrin regulates tissue specialization and clonal expansion of lung germinal center and memory B cells after viral infection
Source: Sci Adv. 2026 Jun 12;12(24):eaeb7633. doi: 10.1126/sciadv.aeb7633 (PMC13262626; doi:10.1126/sciadv.aeb7633)
Supplement: Supplementary file 1 — Figs. S1 to S8 [file sciadv.aeb7633_sm.pdf]

Science Advances
AAAS

Supplementary Materials for

B cell αv integrin regulates tissue specialization and clonal expansion of lung germinal center and memory B cells after viral infection

Andrea Montiel-Armendariz *et al.*

Corresponding author: Mridu Acharya, mridu.acharya@seattlechildrens.org

Sci. Adv. **12**, eaeb7633 (2026)
DOI: 10.1126/sciadv.aeb7633

This PDF file includes:

Figs. S1 to S8

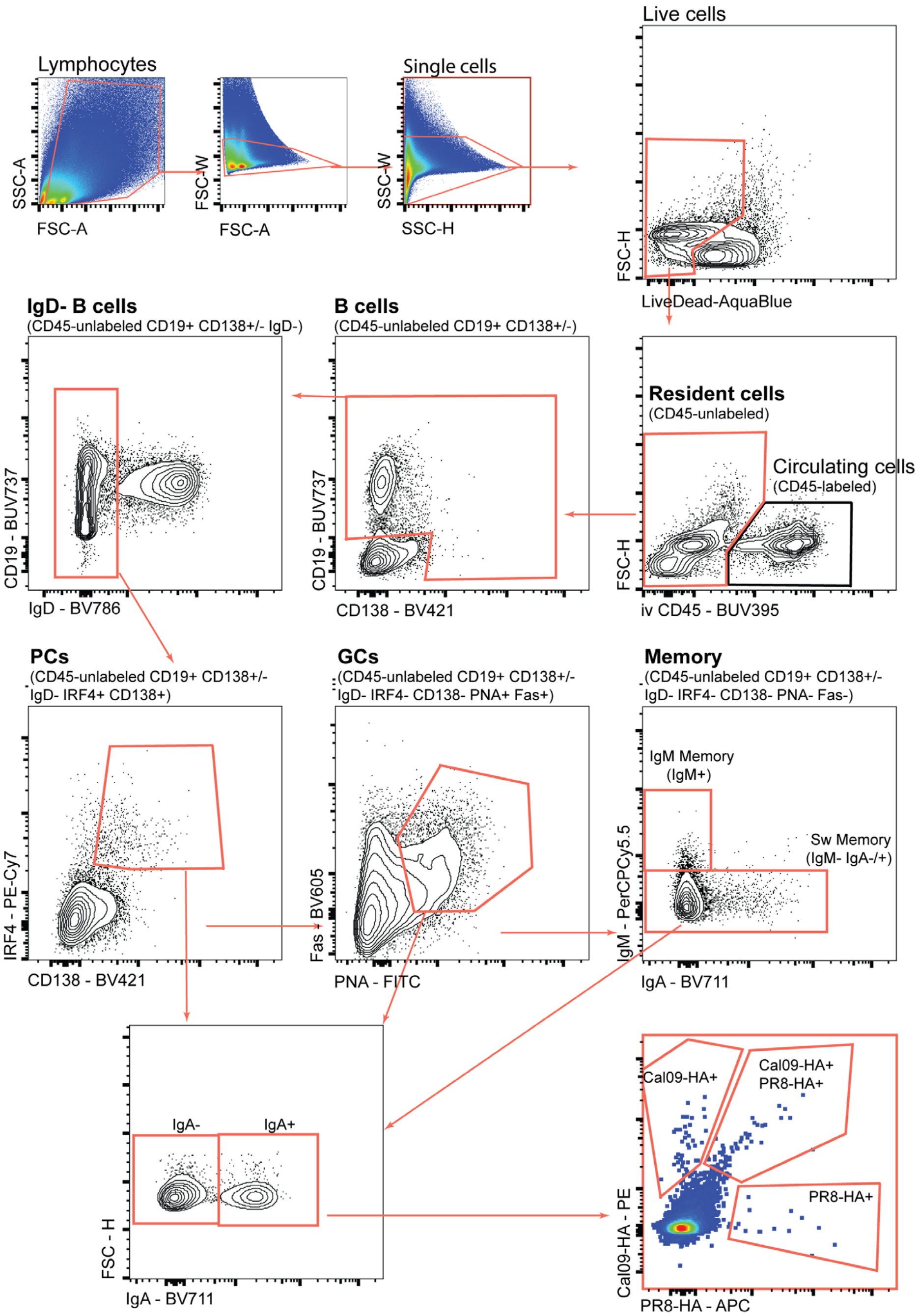

Supplementary Figure 1. *Gating strategy for lung and medLN B cells.*

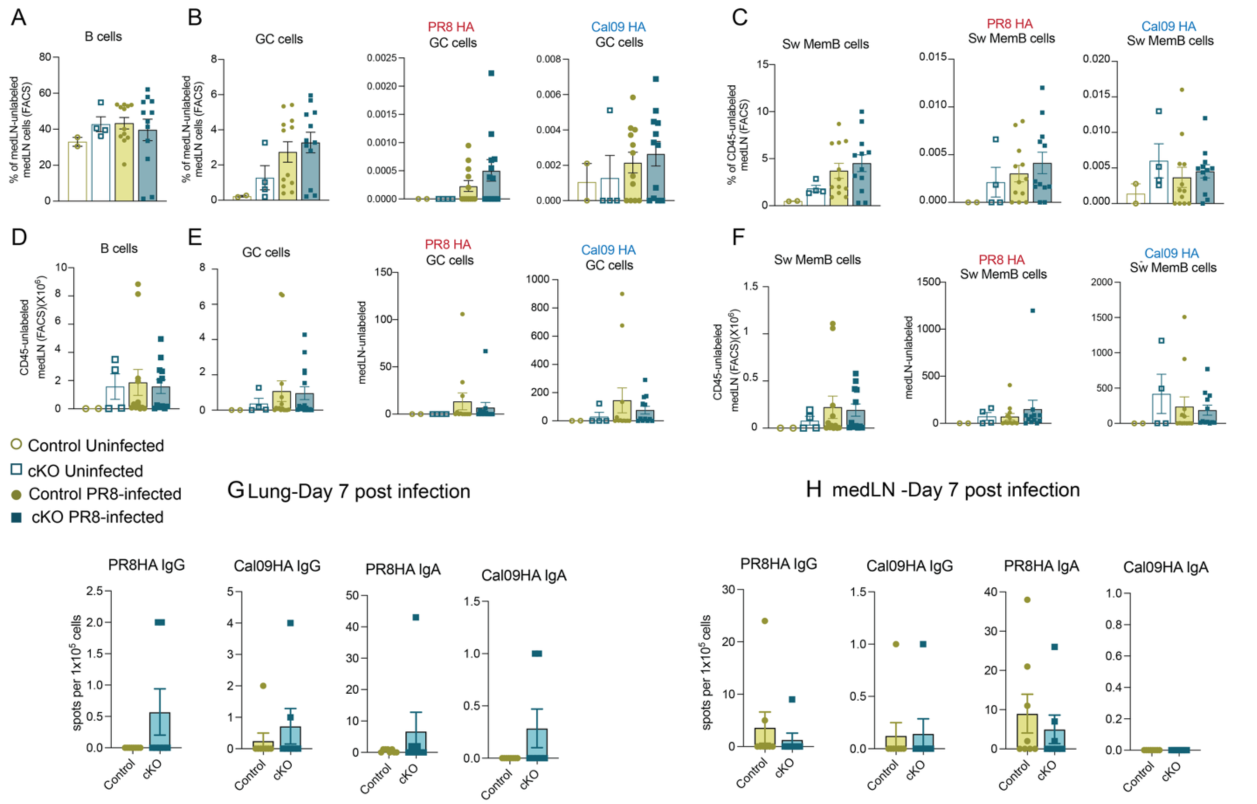

Supplementary Figure 2. *B cell subsets in thel lung and medLN induced by infection***.** $\alpha v^{+/+}$ $CD19^{Cre+}$ (control) and $\alpha v^{fl/fl}$ $CD19^{Cre+}$ (cKO) mice were infected i.n (intranasally). with low dose of live H1N1 PR8 IAV. After 14 days of infection all mice received r.o. injection with 1µg α-CD45-BUV395 five minutes prior to euthanasia, lung and medLN were collected for analysis. (**A**) Quantification of the medLN B cells as a frequency of total resident medLN cells. **(B-C)** Quantification of the frequency of total GC B cells **(B)** or switched memory phenotype B cells **(C)** in the medLN. PR8-HA specific (middle) and Cal09-HA specific (right) within the GC or memory B cells are also shown. **(D-F)** Quantification of medLN B cells as cell numbers for same B cell subsets as in **(A-C)**. **(G-H)** ELISPOT analysis of antibody secreting cells in either lung **(G)** or medLN **(H)** at day 7 post infection. Each dot represents an individual mouse (n= 2-4 mice for uninfected group and n≥ 12mice for infected groups). Data are means ± SEM combined from 2 independent experiments.

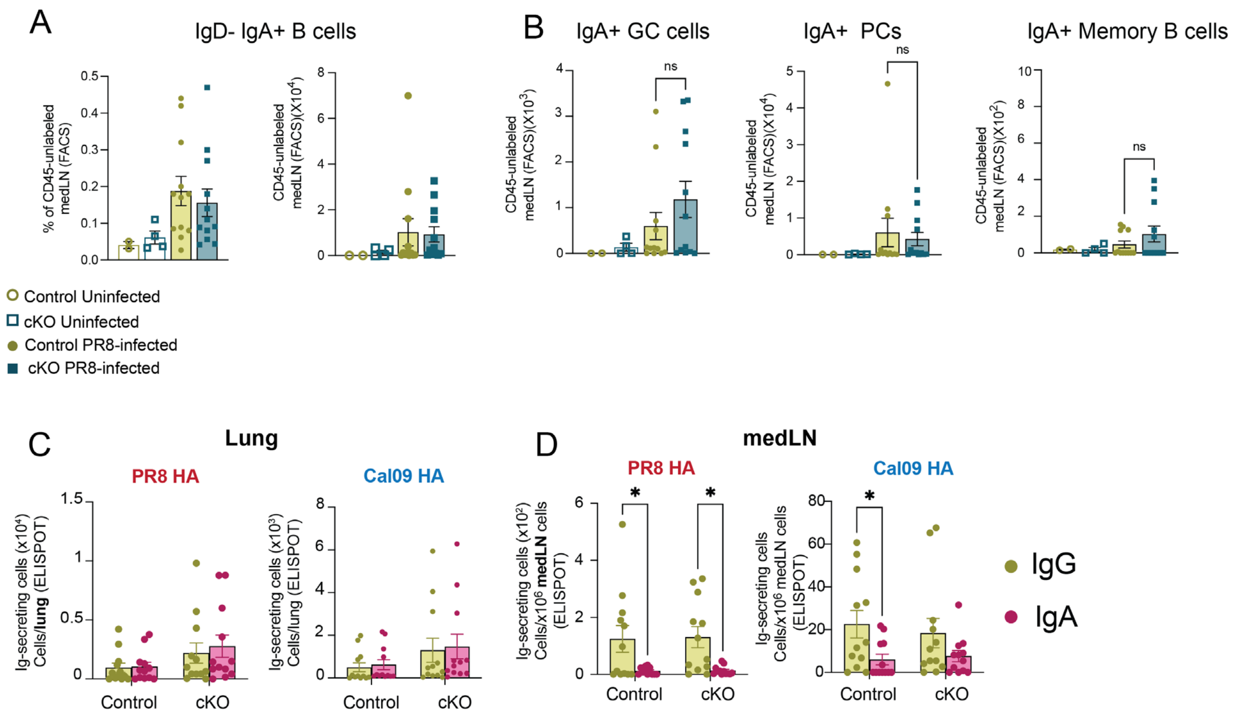

Supplementary Figure 3. *Induction of IgA+ B cells in medLN and lungs after infection.* $\alpha v^{+/+}$ $CD19^{Cre+}$ (control) and $\alpha v^{fl/fl}$ $CD19^{Cre+}$ (cKO) mice were infected i.n. with low dose of live H1N1 PR8 IAV. After 14 days of infection all mice received r.o. injection with 1μg α-CD45-BUV395 five minutes prior to euthanasia, lung and medLN were collected for analysis. **(A)** Quantification of medLN IgD^- IgA^+ B cells by flow cytometry as a frequency of resident medLN cells. **(B)** Analysis of cell numbers for IgA+ B cell subsets identified by flow cytometry in control and cKO mice after infection. Each dot represents an individual mouse (n= 2-4 mice for uninfected group and n≥ 12mice for infected groups). (**C-D**) Comparison of the Ig-secreting cells in the lungs **(B)** or medLN **(C)** that recognize PR8-HA (left) or Cal09-HA (right) as detected by ELISPOT. Data are means ± SEM of 2 experiment from 3 independent experiments in (**A-C)** and combined data from two independent experiments are presented in **D**. *p<0.05 by Mann-Whitney U-test between the two PR8-infected groups.

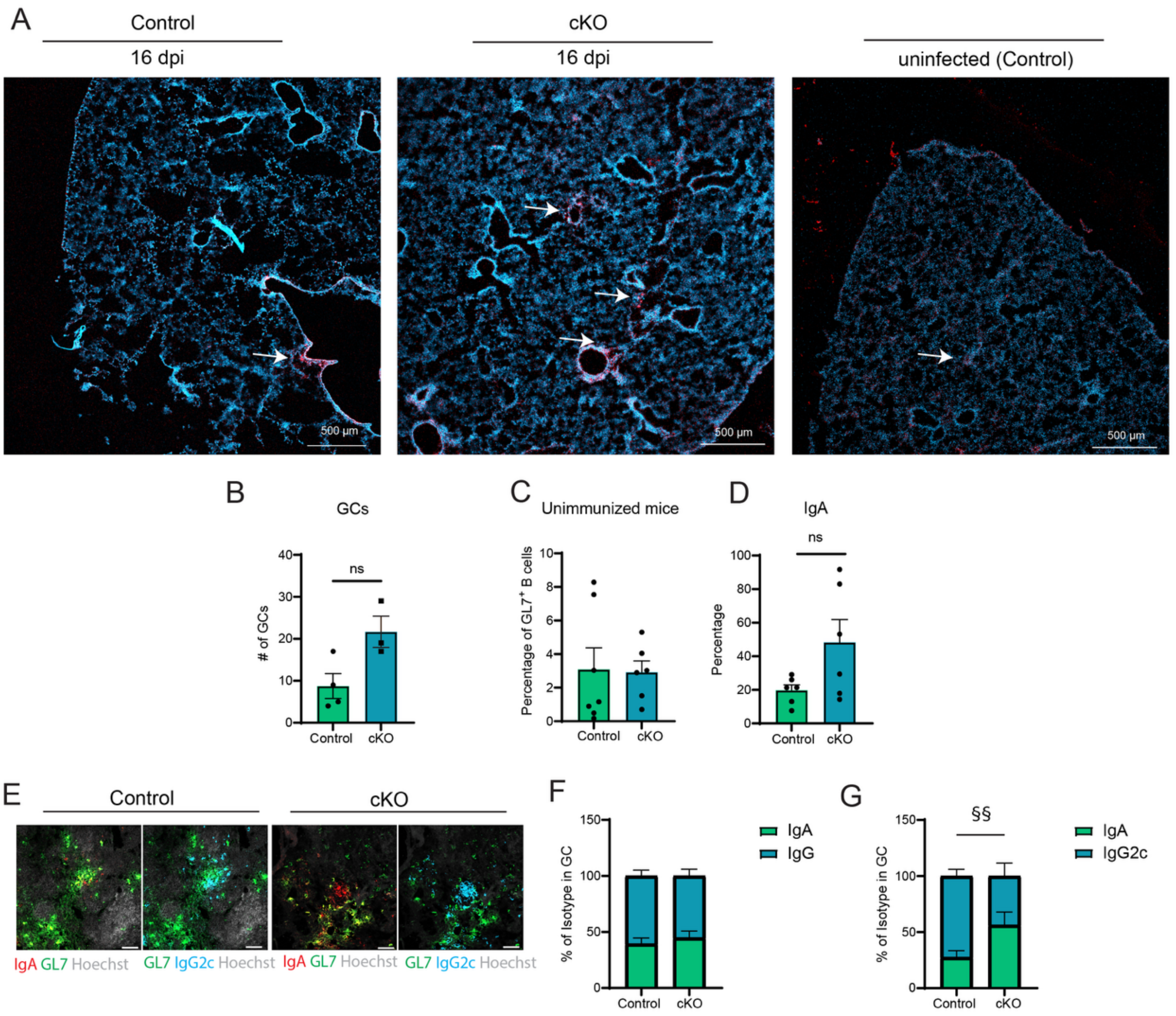

Supplementary Figure 4. *Analysis of GC structures in the lungs.* $\alpha v^{+/+}$ $CD19^{Cre+}$ (control) and $\alpha v^{fl/fl}$ $CD19^{Cre+}$ (cKO) mice were infected i.n. with low dose of live H1N1 PR8 IAV and lung sections were analyzed as in **Fig 3** **(A)**. Expanded and contrast-enhanced images from **Fig 3B** showing IgA expression (red) in lung sections from unimmunized control mouse and lung sections from control and cKO mice 16 days post-infection (dpi). Arrows indicate areas with IgA. **(B-D)** Quantification of immunofluorescence staining in the lung sections showing: **(B**) average number of GCs per mouse lung section**(C)**, percentage of GL7+ B cells overall in lung sections from unimmunized mice, and **(D)** percentage of IgA+ cells relative to total B cells. **(E)** Representative immunofluorescence images of lung sections stained for IgA (red), IgG2c (cyan), GL7 (green), and Hoechst (gray) in control and cKO mice at 16 dpi. **(F-G)** Quantification of the percentage of IgA+ and IgG **(G)** or IgA and IgG2c+ **(H)** areas within the germinal center (GC) at 16 dpi. Data in **(B, D, F)** are pooled from three independent experiments. Data in **(C)** and **(G)** are representative of three independent experiments. Graphs show means ± SEM (N=at lease 3 mice per group). *p < 0.05 by Mann-Whitney test; §§p < 0.001 for IgA expression (control vs. cKO) by two-way ANOVA followed by Tukey's post-hoc test. Scale bar: 50 µm.

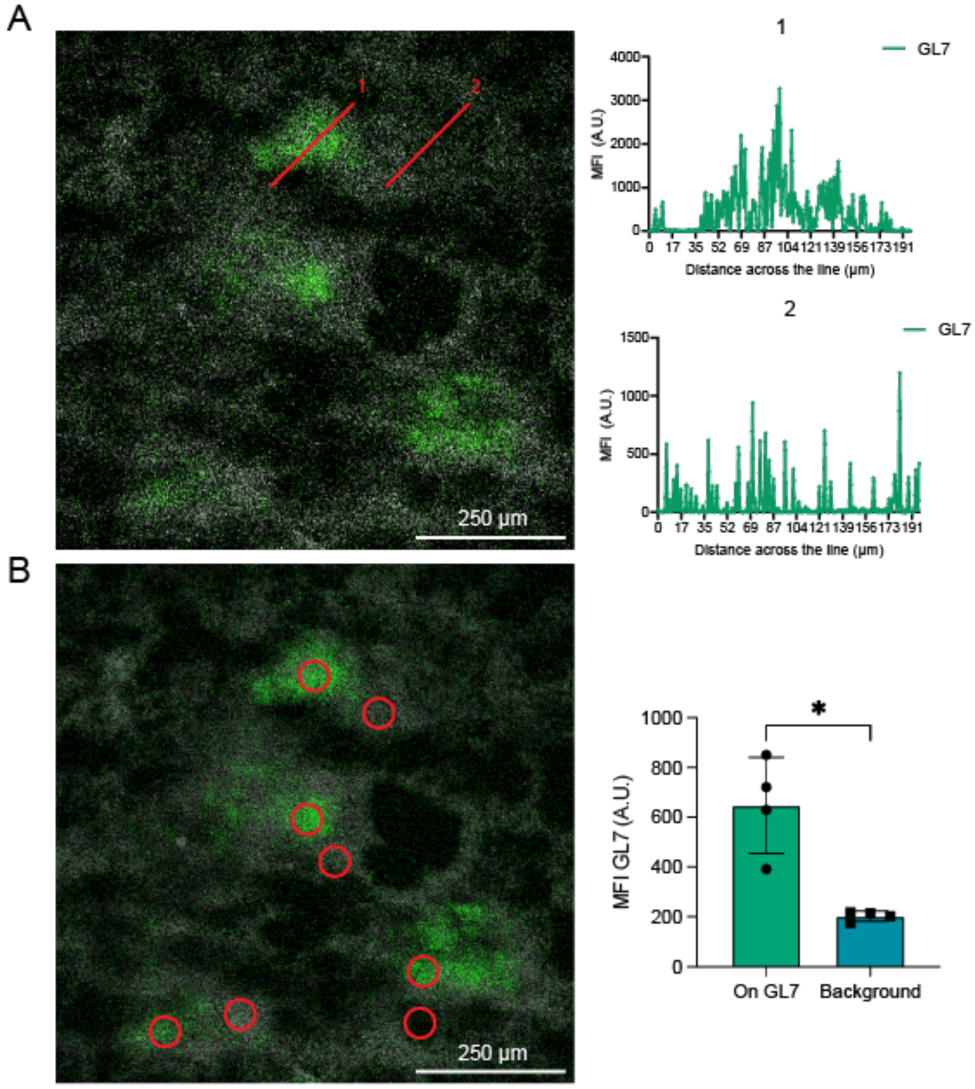

Supplementary Figure 5. *Identification of iBALT-GC areas in the lung using GL7 as a marker for GC cells.* Mice were infected with live PR8 and harvested for analysis of lungs by confocal microscopy as in **Figure 3**. **(A)** Line scan across GL7 positive (1) and negative (2) region. Red lines represent areas delimited as GL7 staining or background. **(B)** Bar graph representing mean and SEM± of mean fluorescence intensity (MFI) of GL7 positive areas compared to background staining. Red circles represent areas delimited as GL7 positive or background. Each dot represent and individual are (GL7 stain or background) in one representative lung section. Scale bar 250 µm. *p<0.05 by Mann-Whitney U-test between the GL7 staining vs background.

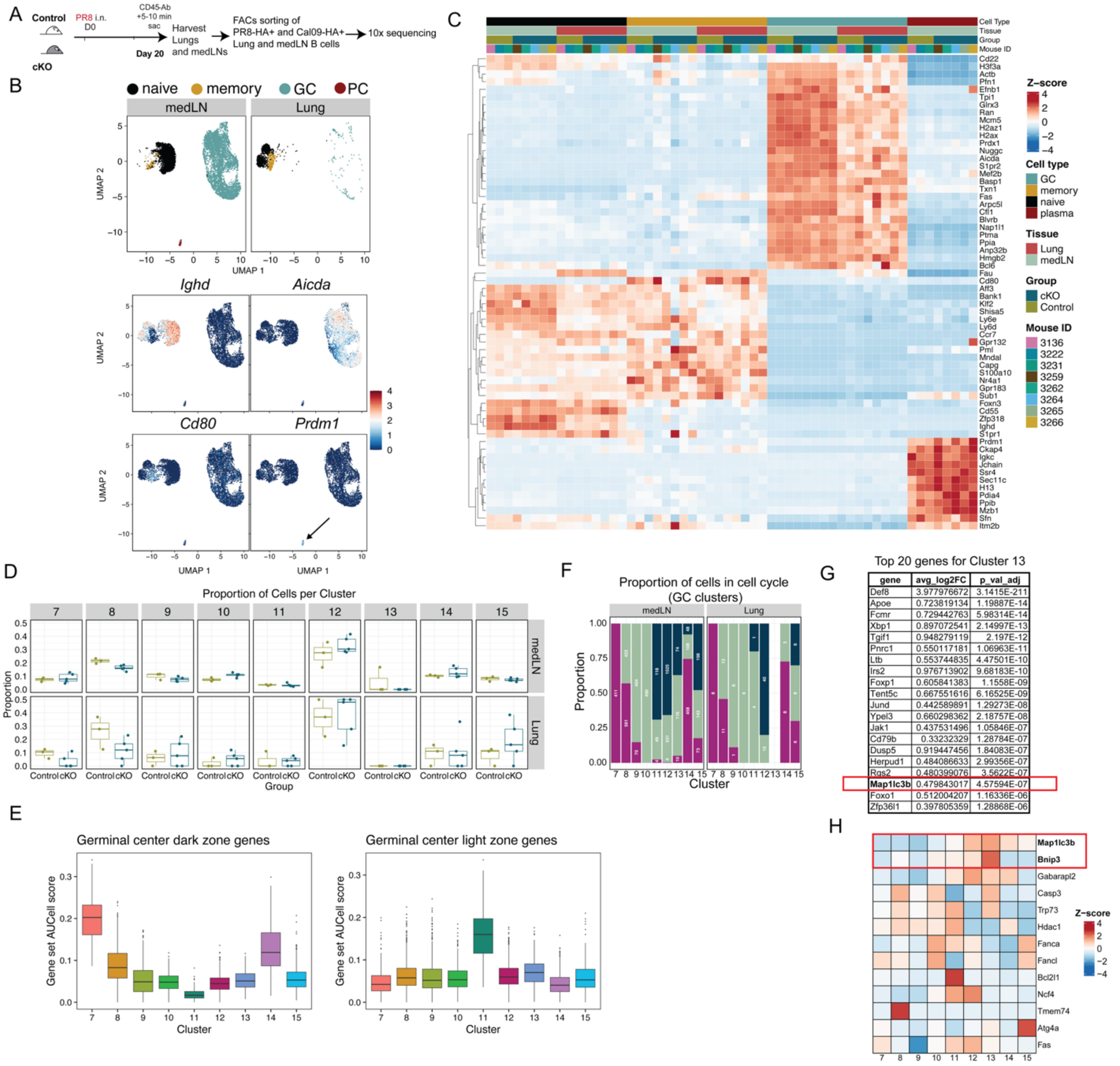

G

Top 20 genes for Cluster 13

gene	avg_log2FC	p_val_adj
Def8	3.977976672	3.1415E-211
Apoe	0.723819134	1.19887E-14
Fcmr	0.729442763	5.98314E-14
Xbp1	0.897072541	2.14997E-13
Tgif1	0.948279119	2.197E-12
Pnrc1	0.550117181	1.06963E-11
Ltb	0.553744835	4.47501E-10
Irs2	0.976713902	9.68183E-10
Foxp1	0.605841383	1.1558E-09
Tent5c	0.667551616	6.16525E-09
Jund	0.442589891	1.29273E-08
Ypel3	0.660298362	2.18757E-08
Jak1	0.437531496	1.05846E-07
Cd79b	0.33232329	1.28784E-07
Dusp5	0.919447456	1.84083E-07
Herpud1	0.484086633	2.99356E-07
Rgs2	0.480399076	3.5622E-07
**Map1lc3b**	0.479843017	4.57594E-07
Foxo1	0.512004207	1.16336E-06
Zfp36l1	0.397805359	1.28868E-06

Supplementary Figure 6. *ScRNA seq on influenza specific lung and medLN B cells from mice infected with PR8.* $\alpha v^{+/+}$ $CD19^{Cre+}$ (control) and $\alpha v^{fl/fl}$ $CD19^{Cre+}$ (cKO) mice were infected with low dose of live PR8 IAV. Lungs and medLN were harvested 20 days post infection. **(A)** Schematic of infection and tissue collection (lung and medLN) for sorting of influenza antigen-specific (PR8HA+ or Cal09HA+) cells. Mice received r.o. injection with α-CD45-BUV395 five minutes prior to euthanasia. **(B)** UMAP representation of scRNAseq data from B cells in lung and medLN. Clusters were assigned to one of four mayor B cell types based on the expression of the following markers: naïve (*Ighd*), GC (*Aicda*), Plasma cells (*Prdm1*) (shown by arrow), and memory (*Cd80*). **(C)** Heatmap of the z-score of canonical markers to differentiate various B cell subpopulations. **(D)** Box plot of the proportion of cells in each GC cluster in medLN (top) and lung (bottom) in the control and cKO mice. Each dot represents one mouse. **(E)** Box plots showing the expression of DZ or LZ gene sets in each GC cluster, quantified by AUCell. **(F)** Quantification of the proportion of cells by cell cycle phase in the GC clusters. **(G)** Table of the top 20 genes with higher expression, as determined by adjusted p value. **(H)** Heatmap of the expression of GC autophagy-related genes.

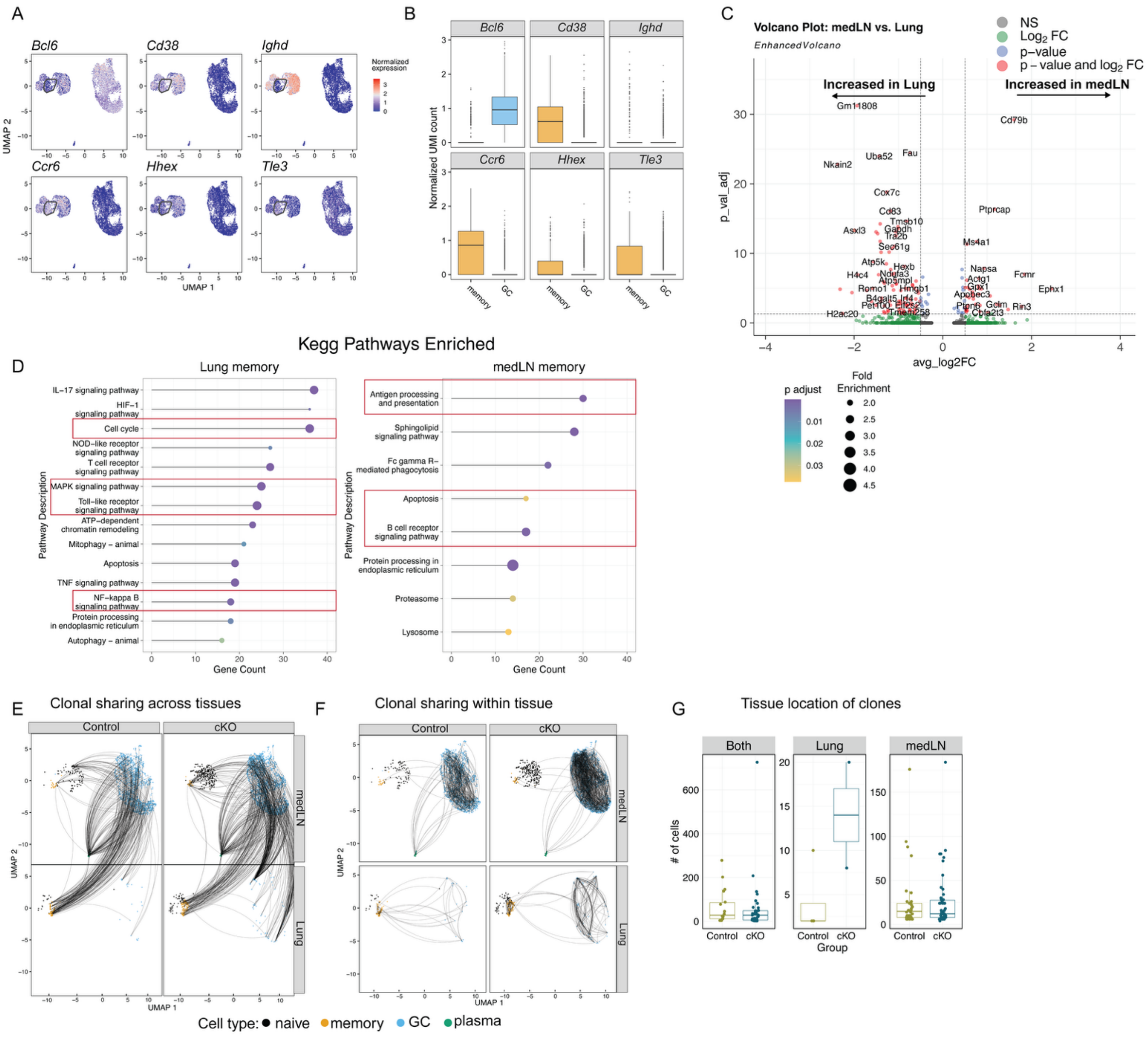

Supplementary Figure 7. *ScRNAseq on influenza specific lung and medLN memory B cells from mice infected with PR8***.** $\alpha v^{+/+}$ $CD19^{Cre+}$ (control) and $\alpha v^{fl/fl}$ $CD19^{Cre+}$ (cKO) mice were infected with low dose of live PR8 IAV. Lungs and medLN were harvested 20 days post infection, as above, **Supp Fig 6. (A**) UMAP showing the expression of genes characterizing memory B cells and other B cell subsets; memory B cells are indicated by the black polygon. **(B)** Box plots showing expression of characteristic B cell subset genes in memory versus GC populations. **(C-D)** Volcano plot comparing the genes highly expressed **(C)** and lollipop plot of the enriched KEGG pathways **(D)** in the lung memory (left) or the medLN memory (right). Lung memory was obtained by grouping clusters 0, 1 and 2-predominantely found in the lungs- and cluster 3 and 4-predominantely found in the medLN. **(E-F)** UMAP representation of the clonal sharing analysis comparing clones shared across tissues **(E)** within same tissue **(F)** in the medLN and lungs of control and cKO groups. Each dot represents a cell, each color represents a cell type, and the lines connect the clonal partners in different subpopulations and/or tissues. **(G)** Quantification of the number of GC cells per clone that are present in both tissues, only in the Lung or only in the medLN. Each dot represents a clone.

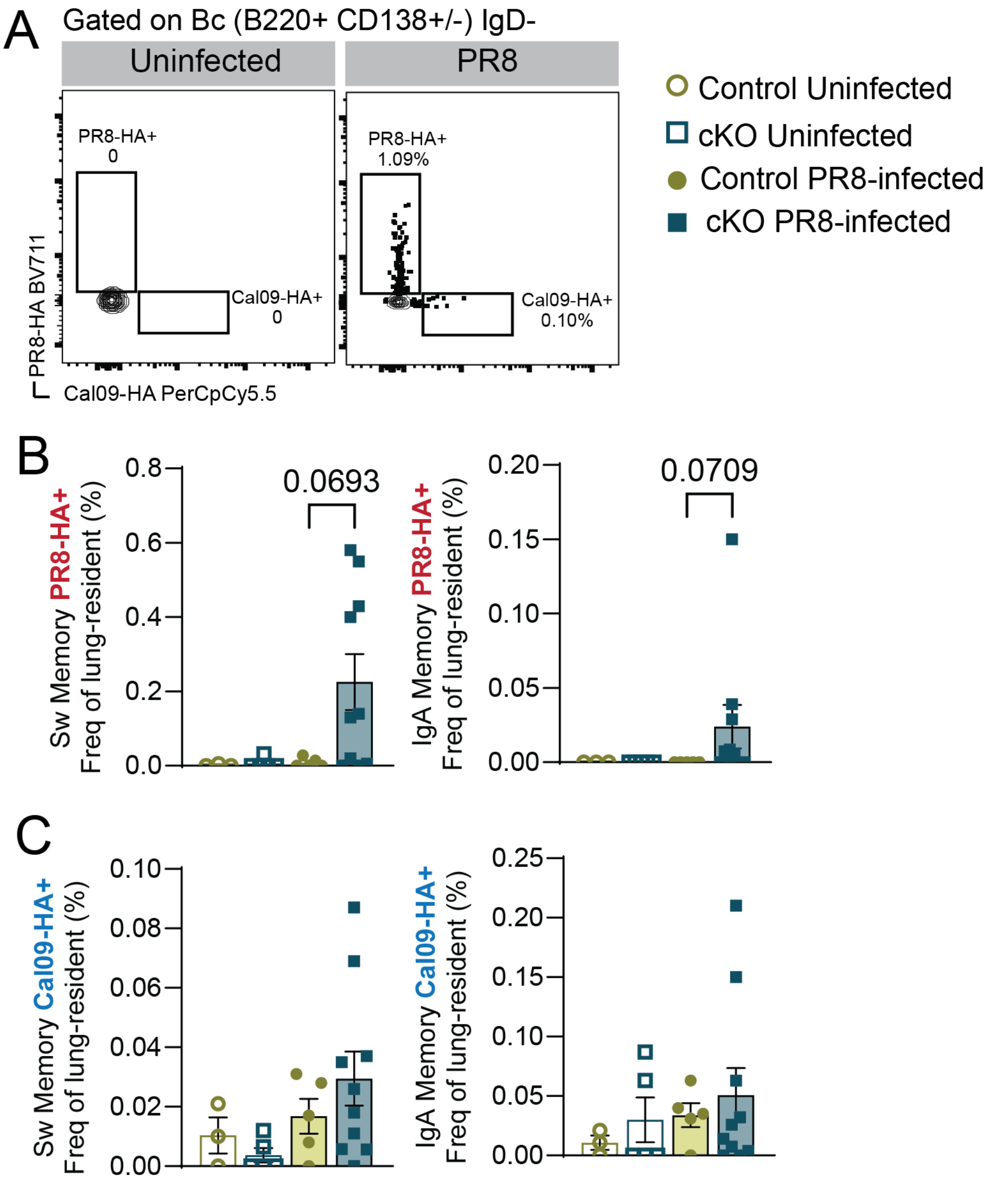

Supplementary Figure 8. *Analysis of lung tissue-resident cross-reactive and antigen specific memory B cells.* $\alpha v^{+/+}$ $CD19^{Cre+}$ (control) and $\alpha v^{fl/fl}$ $CD19^{Cre+}$ (cKO) mice were infected and harvested as in **Fig 6A.** Mice received r.o. injection with α-CD45-BUV395 five minutes prior to euthanasia. **(A)** Representative flow cytometry gates of the PR8-HA – SA-BV711 and Cal09-HA – SA-PerCpCy5.5 tetramers in an uninfected (left) and infected (right) mouse; gated as live resident (CD45-unlabeled, see gating strategy on **Supp Fig1**) B220+ CD138+/- IgD-. **(B-C)** Quantification of the frequency of Sw and IgA memory specific for PR8-HA **(B)** or Cal09-HA **(C)** as identified by flow cytometry. Each dot represents one mouse; data are means SEM± Representative experiment from 3 independent repeats.
